# Diversity of spore morphology among fission yeasts

**DOI:** 10.1371/journal.pone.0355697

**Published:** 2026-08-19

**Authors:** Tomoki Sakaguchi, Yuhei O. Tahara, Makoto Miyata, Taro Nakamura

**Affiliations:** 1 Department of Biology, Graduate School of Science, Osaka Metropolitan University, Sumiyoshi-ku, Osaka, Japan; 2 The OMU Advanced Research Institute for Natural Science and Technology, Osaka Metropolitan University, Sumiyoshi-ku, Osaka, Japan; Tulane University Health Sciences Center: Tulane University School of Medicine, UNITED STATES OF AMERICA

## Abstract

Fungi produce spores as a strategy for survival and dispersal. Spore shape and surface structure are thought to facilitate adhesion to hosts or vectors, and the outermost spore layer is associated with spore hydrophobicity and stress tolerance. Although the morphology of *Schizosaccharomyces pombe* spores has been well characterized—including a distinctive bumpy surface and an outermost proteinaceous layer—spore morphology in other fission yeasts remains poorly understood. Here we compared the structure of spores from four fission yeast species—*S. pombe, S. octosporus, S. japonicus,* and *S. versatilis*—using various microscopy techniques. Optical and electron microscopy revealed that the surface structures of fission yeast spores differ markedly among species. Notably, the fibrillar surface structures observed in *S. pombe* spores are absent in other fission yeast spores. Ultrathin-section electron microscopy showed that the characteristic electron-dense structures of the outermost spore layer in *S. pombe* are also largely absent in the other species. Among the four species examined, *S. pombe* spores exhibited the highest stress tolerance to heat and ethanol. Collectively, these results suggest that fission yeasts have acquired distinct spore morphologies, which may have contributed to their adaptation to diverse habitats.

## Introduction

Fungi produce spores, including conidia and ascospores, whose morphology and surface chemical properties play important roles in survival and dispersal [1,2]. Fungal spores exhibit considerable morphological diversity. For example, the spores of *Saccharomyces cerevisiae* are spherical with a ridged surface [3,4], those of *Ashbya gossypii*, *Eremothecium ashbyi*, *Nematospora coryli*, *Eremothecium cymbalariae*, and *Holleya sinecauda* are needle-like in shape [5], while the fission yeast *Schizosaccharomyces pombe* produces spores with a characteristic bumpy surface [6].

The outermost layer of spores is important in determining their surface chemical properties. In certain ascomycete and basidiomycete species, spores are coated with hydrophobins—proteins that confer hydrophobicity and facilitate interactions such as adhesion to hosts [7]. However, hydrophobins are not conserved in yeasts; in the genera *Saccharomyces* and *Kluyveromyces*, for example, spores are instead covered by a dityrosine layer [8,9], which is essential for high stress resistance [10]. The *S. pombe* spore wall is also considered to comprise polysaccharide layers, similar to those of *S. cerevisiae*; however, its outermost layer is markedly different, consisting of a single protein, Isp3 [11,12]. Similar to the dityrosine layer in *S. cerevisiae* spores, the “Isp3 layer” in *S. pombe* spores is essential in conferring stress resistance [12].

At present, six species are recognized within the genus *Schizosaccharomyces*: *S. pombe*, *S. japonicus*, *S. octosporus*, *S. cryophilus*, *S. osmophilus*, and *S. lindneri* [13]. However, recent phylogenetic and genomic analyses have suggested that two *S. japonicus*, variants, *S. japonicus* var. *japonicus* and *S. japonicus* var. *versatilis*, may represent distinct species [14,15]; in this study, therefore, we refer to them as *S. japonicus* and *S. versatilis*. The four species *S. octosporus*, *S. cryophilus*, *S. osmophilus*, and *S. lindneri* are phylogenetically closely related (S1 Fig): the vegetative cells of *S. octosporus*, *S. osmophilus*, and *S. lindneri* are morphologically indistinguishable, while those of *S. cryophilus* are slightly smaller [13,16,17]. During sexual reproduction, these species, together with *S. octosporus*, *S. japonicus*, and *S. versatilis*, produce up to eight spores within a single ascus, whereas *S. pombe* produces only four spores [13,16–20].

Despite the known similarities in vegetative morphology and reproductive characteristics, little is known about interspecies differences in spore morphology and surface structure among the wider fission yeasts. Previous studies using ultrathin-section electron microscopy have reported morphological changes during sporulation and in ascus spores in *S. pombe, S. octosporus*, and *S. japonicus* [21,22]; however, the detailed ultrastructure of individual free spores has not been fully characterized. In this study, therefore, we have systematically analyzed the morphology and surface structures of free spores from four fission yeast species—*S. pombe*, *S. octosporus*, *S. japonicus*, and *S. versatilis—*using multiple electron microscopy techniques. In addition, we have compared spore stress resistance to ethanol and heat.

## Results

### Optical microscopy of fission yeast spores

**Fig 1:**
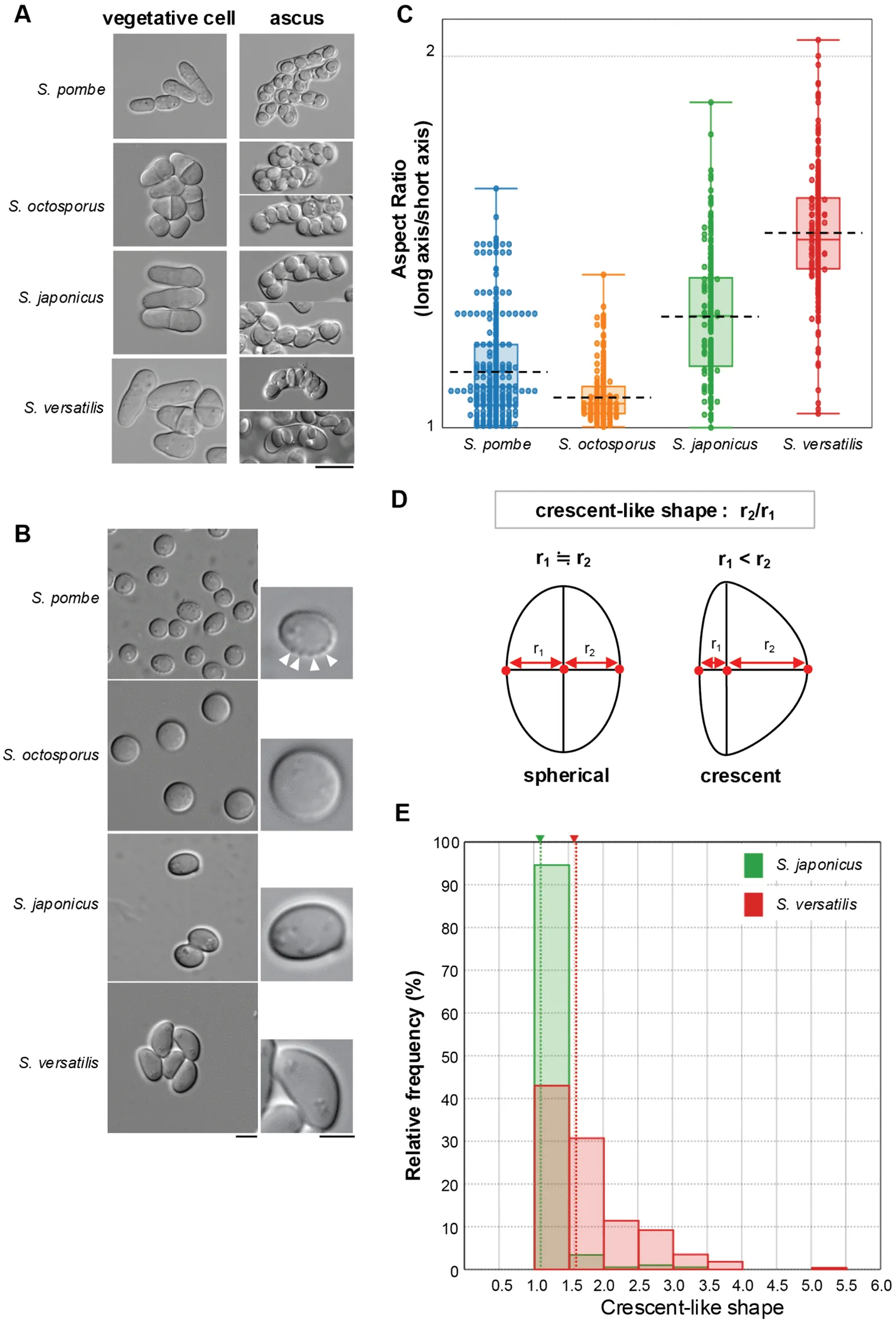
Comparison of spore shapes among the four fission yeast species. (A) Vegetative cells and ascospores of *S. pombe*, *S. octosporus*, *S. japonicus*, and *S. versatilis*. Scale bars, 10 µm. (B) Left, shapes of the spores of four fission yeasts. Right, magnified images of a single spore from each species. White arrowheads indicate the dot-like structure on the spore periphery. Scale bars, 4 µm. (C) Box-plot of the aspect ratio of spores from each species (n > 200). Individual data plots and means (black dotted lines) are presented as a box-plot. (D) Definition of the crescent-shaped spore. (E) Frequency of crescent-shape spores of *S. japonicus* and *S. versatilis* (n > 200). Arrowheads and dotted lines indicate median values.

To determine whether spore morphology differs among *S. pombe, S. octosporus*, *S. japonicus*, and *S. versatilis*, we first performed optical microscopy. As reported previously [18–20], these four species of fission yeast exhibited different morphology in both vegetative cells and asci, with *S. pombe* having the smallest vegetative cells (Fig 1A) [20]. Differential interference contrast microscopy of spores from each species revealed dot-like structures along the outline of *S. pombe* spores (Fig 1B), corresponding to the characteristic bumpy surface of *S. pombe* spores [6], whereas the spores of the other species were smooth in outline (Fig 1B). These observations suggest that spore surface structures differ between *S. pombe* and the other three fission yeasts.

As described in previous studies [18,19,23,24], spherical spores were frequently observed in *S. pombe* and *S. octosporus*, whereas elliptical spores were predominantly found in *S. japonicus* and *S. versatilis* (Fig 1B). Consistent with these microscopic observations, the aspect ratio (AR) of *S. pombe* and *S. octosporus* spores was close to 1 (1.15 ± 0.12 and 1.08 ± 0.06, respectively), indicating a predominantly spherical morphology (Fig 1C). In contrast, the AR of *S. japonicus* and *S. versatilis* was much greater than 1 (1.30 ± 0.16 and 1.52 ± 0.16, respectively), suggesting a more elliptical shape. *S. pombe* spores also exhibited polymorphism in shape, ranging from spherical to elliptical, as reflected in the wide range of AR values (Fig 1C). In contrast, *S. octosporus* spores showed little variation, with nearly all spores exhibiting a uniform spherical morphology and a narrow AR range. Although *S. japonicus* spores exhibited a wide range of AR values (Fig 1C), it is likely that some spores that appeared spherical were actually elliptical spores viewed along the short axis. Therefore, the actual AR values of *S. japonicus* spores may be underestimated.

As previously reported [19], *S. versatilis* exhibited a high proportion of free spores with a crescent-shaped morphology, but these spores were rarely observed in *S. japonicus* (Fig 1B). To quantify this observation, we defined a parameter for the “crescent-like” shape as the ratio of the distances from the intersection of the long and short axes to each end of the short axis (Fig 1D), and determined the frequency of crescent-like spores in the two subspecies *S. japonicus* and *S. versatilis* (Fig 1E). Nearly 95% of spores had a crescent-like shape value of <1.5 in *S. japonicus* (Fig 1E, green) whereas more than 50% of spores exhibited crescent-like shape values >1.5 *S. versatilis* (Fig 1E, red). Moreover, the median value of the crescent-like shape parameter was higher in *S. versatilis* than in *S. japonicus* (Fig 1E, arrowheads and dotted lines), indicating that *S. japonicus* spores are more elliptical, whereas *S. versatilis* spores are more crescent-shaped. This observation confirms that spore morphology differs substantially between *S. japonicus* and *S. versatilis*, further supporting their classification as distinct species [14,15].

### Quick-freeze deep etch electron microcopy of fission yeast spores

**Fig 2:**
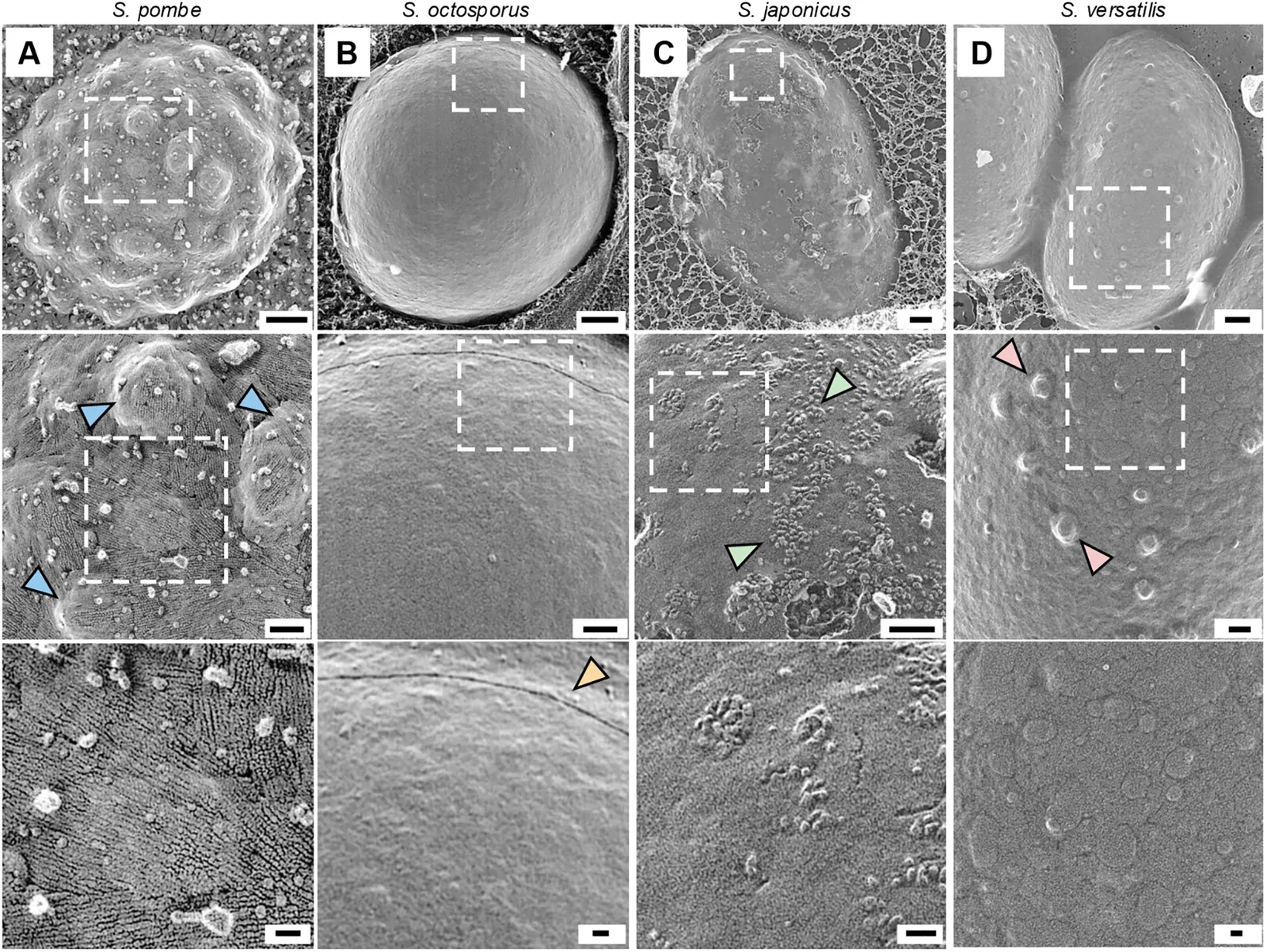
QFDE-EM observation of the spore surface of the four fission yeast species. (A-D) Upper panels, overall images of spores from *S. pombe* (A), *S. octosporus* (B), *S. japonicus* (C), and *S. versatilis* (D). Scale bars, 400 nm. Middle panels, magnified images of the boxed regions in upper panels. Arrowheads indicate characteristic structures on the spore surface. Scale bars, 120 nm. Lower panels, magnified images of the boxed regions in the middle panels. Arrowheads indicate the outermost spore layer. Scale bars, 40 nm.

Next, we examined the surface structures of spores from each species in greater detail using quick-freeze deep-etch electron microscopy (QFDE-EM), which has previously been applied to high-resolution, three-dimensional imaging of the spore structure of *S. pombe* [25]. Due to rapid freezing, samples are imaged in a state that closely resembles their native condition. Consistent with our optical microscopy observations, *S. octosporus* and *S. pombe* exhibited spherical spores, *S. japonicus* had elliptical spores, and *S. versatilis* produced crescent-shaped spores (Fig 2).

Also consistent with optical microscopy, the characteristic bumpy surface structure of *S. pombe* spores [6] was clearly observed by QFDE-EM (Fig 2A, arrowheads), whereas the surface of the other spores was smooth (Figs 2B–D). *S. octosporus* spores displayed no notable surface structures, but were covered with a thin outer layer (Fig 2B, arrowhead). Interestingly, the surface of *S. japonicus* spores displayed a distinct granular texture (Fig 2C, arrowheads); this feature was observed in most spores, indicative of a genuine surface structure rather than an artifact. Although no dot-like structures were detected on *S. versatilis* spores by optical microscopy, QFDE-EM revealed that the spore surface, although essentially smooth, was punctuated by small, scattered bumps (Fig 2D, arrowheads).

### Parallel plasma membrane invaginations are conserved among fission yeast spores

**Fig 3:**
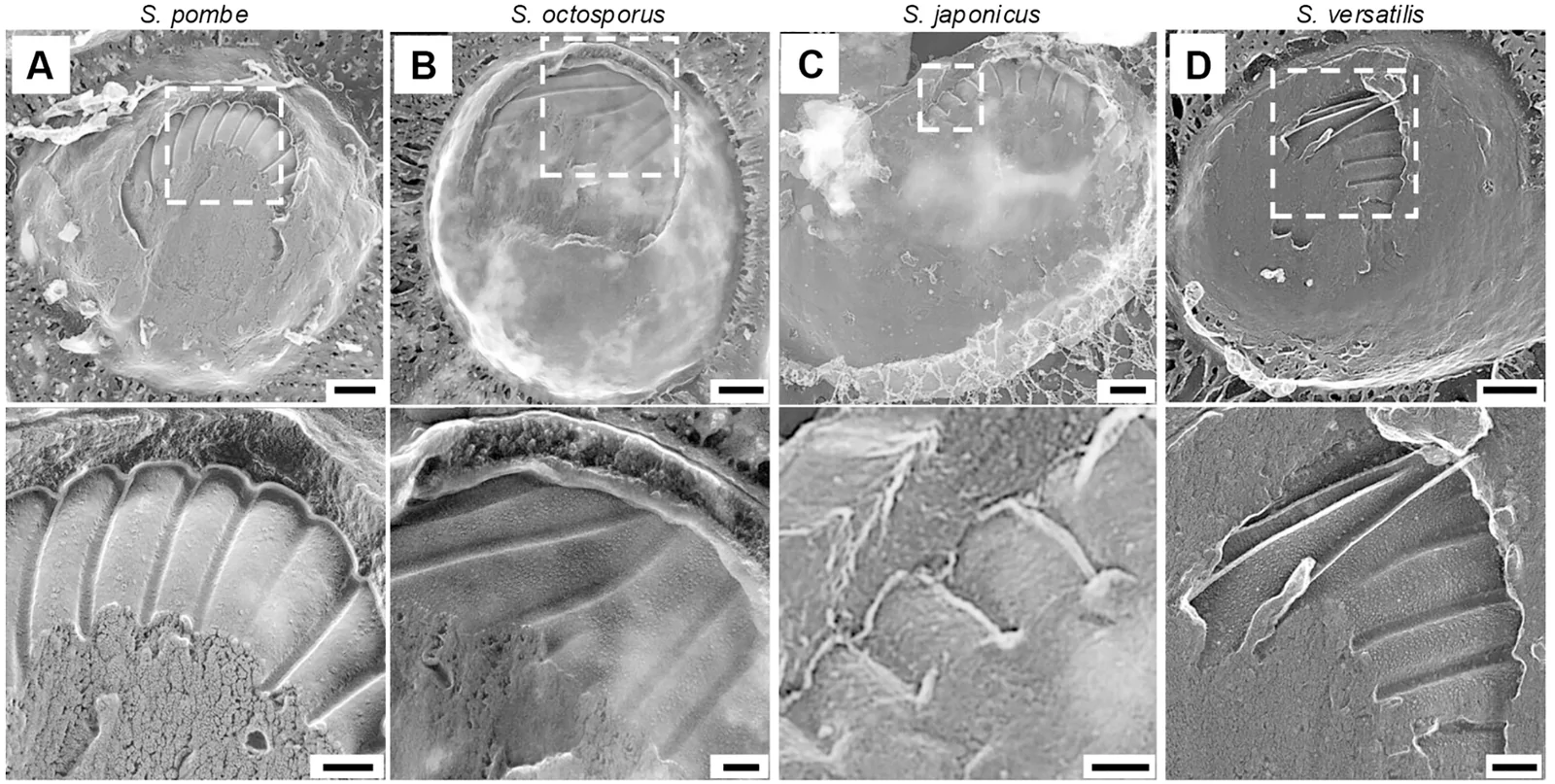
QFDE-EM observations of invaginations on the spore membrane of the four fission yeast species. (A-D) Upper panels, fractured spores of *S. pombe* (A), *S. octosporus* (B), *S. japonicus* (C), and *S. versatilis* (D). Scale bars, 400 nm. Lower panels show magnified images of the boxed regions in the upper panels. Scale bars, 120 nm.

The spores of *S. pombe* exhibit characteristic invaginations aligned in parallel on the plasma membrane—structures that are not observed in vegetative cells [25]. We therefore investigated whether similar invaginated structures are present on the spore plasma membranes of other fission yeast species by QFDE-EM. Notably, the invaginated structures were oriented in parallel in all four species and their morphology was similar (Fig 3). These findings indicate that, whereas the external structures of spores vary significantly among species, the presence of parallel plasma membrane invaginations is conserved across fission yeasts.

**Fig 4:**
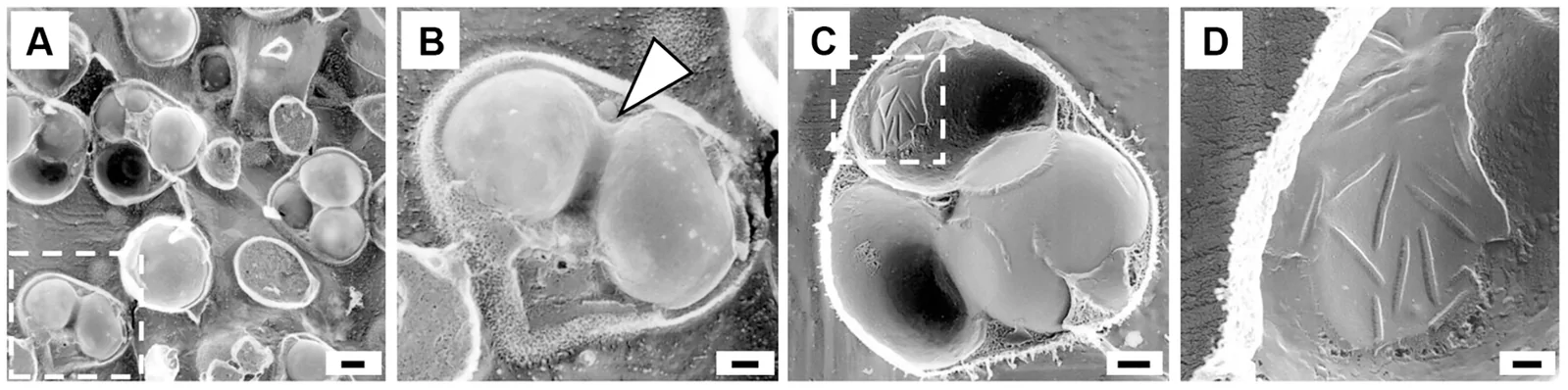
QFDE-EM observations of *S. cerevisiae* spores. (A) Field image of *S. cerevisiae* spores. Scale bar, 1 µm. (B) Magnified image of the single ascus boxed in A. Arrowhead indicates the interspore bridge of the ascospore. Scale bar, 400 nm. (C) Ascus containing a spore with a fractured spore wall. Invagination structures on the spore membrane are partially exposed by the fracture. Scale bar, 400 nm. (D) Magnified images of the boxed region in C. Scale bar, 120 nm.

To determine whether the invaginated structures are broadly conserved among fungal spores, we also analyzed spores of the budding yeast *S. cerevisiae* (Fig 4). Because *S. cerevisiae* spores do not naturally dissociate from the ascus, they are typically isolated by enzymatic digestion of the ascus wall [4]. To eliminate any possible effects of enzymatic treatment on the spore surface, we observed asci directly by QFDE-EM without digestion. In the sample preparations, many cells with partially disrupted asci were observed, allowing direct visualization of the enclosed spores (Fig 4A). In some samples, interspore bridges previously observed by conventional scanning electron microscopy [3] were also observed by QFDE-EM (Fig 4B, arrowhead). Notably, membrane invaginations were observed in a subset of spores with partially exposed plasma membranes (Figs 4C and D); however, the invaginated structures appeared shorter and more randomly oriented, resembling the eisosomes found in vegetative *S. cerevisiae* cells [26]. These observations suggest that the characteristics of the spore plasma membrane may differ substantially between fission yeasts and other yeast genera.

### The outermost spore layer differs among fission yeasts

**Fig 5:**
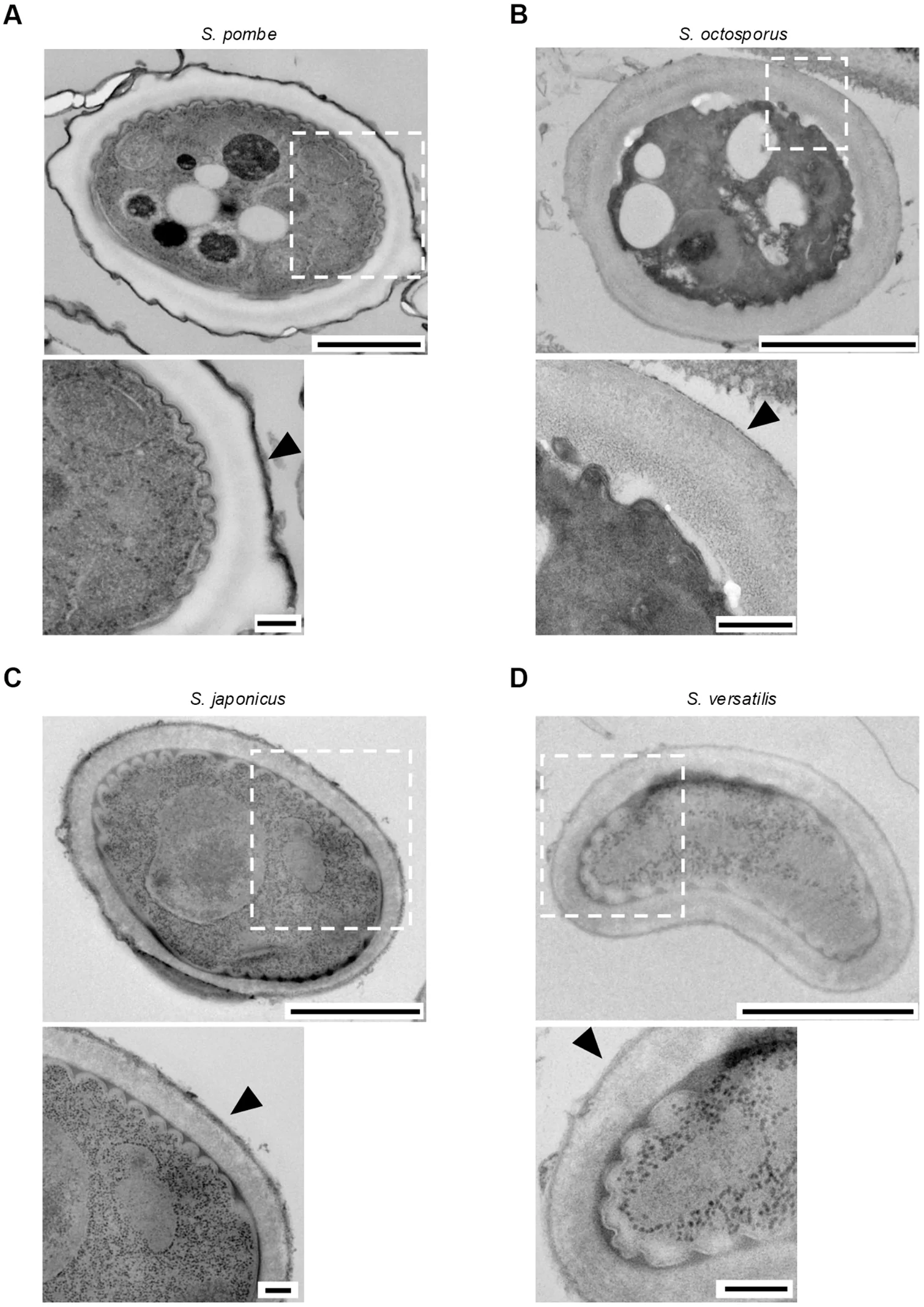
Spores of the four fission yeast species observed by ultrathin-section electron microscopy. (A-D) Upper panels, overall images of spores from *S. pombe* (A), *S. octosporus* (B), *S. japonicus* (C), and *S. versatilis* (D). Scale bars, 1 µm. Lower panels, magnified images of boxed regions in the upper panels. Black arrowheads indicate the outermost layer. Scale bars, 200 nm.

Our previous QFDE-EM analysis demonstrated the presence of a fibrillar structure on the surface of *S. pombe* spores, corresponding to the Isp3 protein layer that coats *S. pombe* spores (Fig 2A) [25]. In contrast, such fibrillar structures were not detected on the spore surfaces of the other fission yeast species (Figs 2B–D). We therefore examined whether structural differences also exist in the outermost spore layer by using ultrathin-section transmission electron microscopy. In *S. pombe*, a distinct electron-dense layer was observed at the outermost region of the spore (Fig 5A, black arrowhead) [12]. As previously demonstrated [12], this electron-dense outermost layer corresponds to the fibrillar surface structures observed by QFDE-EM (Fig 2A, lower panel). In contrast, the outermost layers of spores from other fission yeasts appeared less electron-dense than that of *S. pombe* (Figs 5B–D, black arrowheads). Although ultrathin-section electron microscopy revealed an electron-dense layer in *S. octosporus* spores, this layer was thinner than that observed in *S. pombe* (Fig 5B). Collectively, these findings demonstrate that the outermost spore layer differs in fission yeast, at least between *S. pombe* and the three other fission yeasts examined herein.

### Interspecies variation in spore stress resistance among the fission yeasts

The outermost layer of spores is known to play a critical role in conferring stress resistance. In particular, the dityrosine layer in *S. cerevisiae* and the Isp3 layer in *S. pombe* have been implicated in enhancing spore resistance to various stresses [10,12]. As our above observations indicated that other fission yeast species possess distinct outermost spore layers, we hypothesized that their spore stress resistance would also differ. We therefore compared the stress resistance of spores from four fission yeast species.

As previous studies have reported mating rates of less than 50% for *S. octosporus* and *S. japonicus* [20,27], we first optimized the sporulation conditions to improve spore formation efficiency. Initially, *S. octosporus* exhibited a mating rate of ~30% at 30°C for 3 days, accompanied by a high proportion of dead cells and asci that failed to form spores properly. Because *S. cryophilus*, a close relative of *S. octosporus*, grows optimally at lower temperatures [16], we next induced sporulation at 15°C, 20°C, 25°C, and 30°C and compared mating rates among the four species (S2A Fig).

Whereas the mating rates of *S. pombe* and *S. japonicus* were largely unaffected by temperature, that of *S. octosporus* increased with decreasing temperature, reaching ~70% at both 20°C and 15°C. *S. versatilis* exhibited the highest mating rate (~70%) at 30°C, with a gradual decline at lower temperatures. Based on these results, subsequent sporulation of *S. octosporus* was performed at 20°C, whereas sporulation of *S. japonicus* was maintained at 30°C. Additional experiments using different sporulation media revealed no substantial temperature-dependent changes in mating rates for *S. pombe*, *S. japonicus*, or *S. versatilis* (S2B Fig).

**Fig 6:**
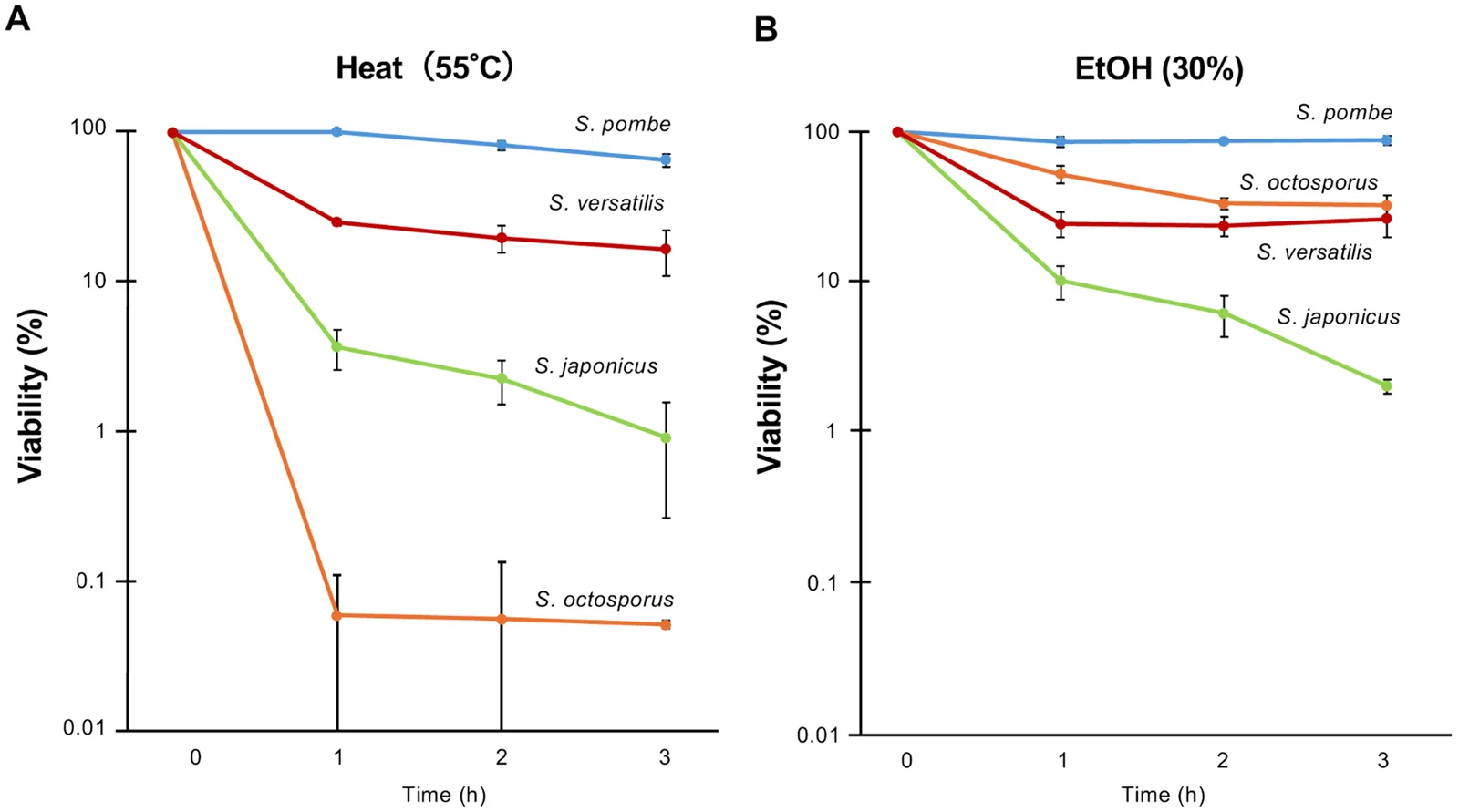
Stress sensitivity of spores from four fission yeast species. (A, B) Stress sensitivity of spores to heat (A) and ethanol (B). Spores from each species were exposed to either heat (55 °C) or ethanol (30%) stress. Spores were plated on YE agar medium and their viability was assessed. Each sample was measured in triplicate, and the mean ± standard deviations (SD) was calculated.

To test stress resistance, we subjected spores of each species to heat and ethanol for 1–3 hours and measured their viability (Figs 6A and B). *S. pombe* spores exhibited the highest resistance to both stresses, while *S. versatilis* spores were more resistant than *S. japonicus* spores under both conditions. Notably, *S. octosporus* spores were highly sensitive to heat stress but showed strong resistance to ethanol stress. Collectively, these results indicate that spore stress resistance varies not only among species but also, in the case of *S. octosporus*, depending on the type of stress applied. Taken together, these findings suggest that the outermost spore layers of *S. pombe*, *S. octosporus*, *S. japonicus*, and *S. versatilis* are structurally distinct and correlates with stress resistance.

## Discussion

In this study, we examined the spore morphology of four *Schizosaccharomyces* species in detail using optical and electron microscopy. We found marked interspecies differences in both spore morphology and stress tolerance. Specifically, *S. octosporus* spores are spherical with a smooth surface, lacking the characteristic structures seen in *S. pombe* spores, while the spores of *S. japonicus* and *S. versatilis* are both oval, although *S. versatilis* spores display a more crescent-like shape. QFDE-EM further revealed that *S. japonicus* spores possess granular surface structures, whereas *S. versatilis* spores exhibit multiple protrusions on their surface. Collectively, these findings demonstrate that spore morphology is highly diverse among fission yeasts. In addition, the pronounced morphological differences between the spores of *S. japonicus* and *S. versatilis* are consistent with recent genomic and genetic studies identifying these yeasts as distinct species [14,15].

Electron microscopy analyses revealed that the outermost spore layer differs considerably between *S. pombe* and the other three species examined. *S. pombe* spores are covered by a single protein, Isp3, which is visualized as an electron-dense layer in transmission electron microscopy and as fibrillar structures in QFDE-EM [12,25]. Such fibrillar structures were not observed on the spores of the other three species, and the electron density of their outermost layer was lower than that of *S. pombe* spores (Figs 2 and 5). The Isp3 layer of *S. pombe* spores can be extracted by simultaneous treatment with SDS and β-mercaptoethanol, and contains a single protein, as detected by SDS–PAGE [11]. By contrast, the other fission yeast species lack genes encoding proteins highly homologous to Isp3 (S1 and S2 Tables). Extraction of the outermost layer of *S. octosporus* spores also yielded two protein bands on SDS–PAGE; however, the bands were both weaker and of different molecular sizes than the *S. pombe* Isp3 protein (S3 Fig). Consistent with this observation, ultrathin-section electron microscopy revealed a thin electron-dense layer in *S. octosporus* spores, suggesting that the spore surface may be covered by a protein distinct from Isp3. In contrast, no distinctive protein bands were detected in *S. japonicus* or *S. versatilis* spores subjected to the same extraction procedure (S3 Fig). Taken together, these results suggest that, unlike *S. pombe*, the spores of *S. japonicus* and *S. versatilis* are not covered by a proteinaceous outer layer. Whereas *S. pombe* typically produces four spores per ascus, *S. octosporus*, *S. japonicus*, and *S. versatilis* usually produce eight spores. However, asci containing only four spores are also occasionally observed in these species. Examination of these spores revealed that none exhibited the bumpy surface characteristic of *S. pombe*. These observations suggest that spore morphology is not related to the number of spores formed within an ascus.

The germination frequencies were similar among *S. pombe* (>90%), *S. japonicus* (88%), and *S. octosporus* (83%) (n > 150), indicating no substantial differences among these species. In contrast, the germination frequency of *S. versatilis* was approximately 20% (n > 150). However, these experiments were performed on YE medium, which may not represent the optimal growth conditions for *S. versatilis*.

Spores of the yeasts *Saccharomyces* and *Kluyveromyces* are covered by a dityrosine layer; however, the genes required for biosynthesis of the dityrosine layer in *S. cerevisiae* are not conserved in *S. pombe*, *S. octosporus*, *S. japonicus*, or *S. versatilis* [12]. Consistent with this, these fission yeast spores did not fluoresce under ultraviolet light, a characteristic feature of the dityrosine layer (S4 Fig). Further studies will be needed to determine the composition of the surface layer of *S. octosporus*, *S. japonicus*, and *S. versatilis* spores.

The spore plasma membrane of *S. pombe* exhibits characteristic invaginated structures that are arranged in parallel and are not observed in vegetative cells [25] (Fig 3A). Here, invaginated structures—also aligned in parallel and with a similar morphology—were observed in the spore plasma membranes of the other three fission yeasts. These observations indicate that, although the external structures of spores vary considerably among fission yeast species, parallel plasma membrane invaginations are conserved among fission yeast spores. In contrast, spores of the budding yeast *S. cerevisiae* have shorter, randomly oriented invaginated structures that are structurally similar to the eisosomes observed in vegetative cells [26,28]. Thus, while membrane invaginations may be broadly conserved across fungi, the genus *Schizosaccharomyces* seems to have acquired a distinct type of structure during evolution. Interestingly, similar parallel invaginated structures have been identified in the red alga *Cyanidioschyzon merolae* [29], which was isolated from hot springs. Because *C. merolae* grows under extreme conditions such as high temperatures (~50 °C) and strong acidity (pH 1–2), it is possible that these parallel invaginated structures contribute to stress tolerance [25].

What, then, are the determinants of spore morphology? In *S. pombe*, the spore wall consists of polysaccharide layers comprising primarily mannan, β-glucan, α-glucan, and a small amount of chitosan, and the Isp3 layer; and its assembly involves numerous genes (reviewed by [30]). The genomes of *S. pombe*, *S. octosporus*, and *S. japonicus* have been sequenced, revealing that ~4000 genes are conserved among fission yeasts, and only 100–400 genes are species-specific [31,32]. Here, database analyses revealed that, with the exception of *isp3*^*+*^, the genes involved in spore wall biosynthesis in *S. pombe* are largely conserved in the other three fission yeast species (S1 Table; [33]), raising the possibility that Isp3 contributes to formation of the characteristic bumpy surface structure in *S pombe* spores. However, spores of the *isp3Δ* mutant still form a bumpy surface [12,25]; thus, the protrusions probably comprise polysaccharides rather than Isp3 itself. We previously demonstrated that Mde10, an ADAM family protein, is involved in forming the characteristic surface of *S. pombe* spores [6]; however, the *mde10 ⁺* gene is conserved in the other three fission yeast species whose spores lack this feature. It is therefore plausible that the genes responsible for species-specific spore shape and surface structure are included within the limited set of 100–400 non-conserved genes in fission yeasts [32].

Our comparison of resistance to heat and alcohol stresses showed that *S. pombe* spores had markedly higher stress tolerance as compared with the other species, possibly attributable to its unique Isp3 layer. It is likely that the spores of the other fission yeasts possess distinct functional properties. For example, the surfaces of filamentous fungal conidia are often hydrophobic, a property that facilitates environmental dispersal and adhesion to hosts [34]. Consistent with this, we found that the spores of *S. octosporus*, *S. japonicus*, and *S. versatilis* adhere much more readily to plastic tips and tubes than *S. pombe* spores (S5 Fig), suggesting higher surface hydrophobicity; *S. versatilis* spores also showed a strong tendency to aggregate and settled in water much more slowly than other spores.

In recent years, *S. japonicus* has attracted attention as a model organism because it exhibits several unique features not observed in *S. pombe*, including hyphal growth, semi-open mitosis [35]. In addition, heterothallic strains have been established in *S. versatilis* and *S. octosporus*, facilitating genetic analyses in these species [20,36]. Together, these advances suggest that future genetic studies may help to elucidate the molecular mechanisms underlying the formation of species-specific spore shapes and surface structures.

Although *S. pombe* has been well studied, its evolutionary history and ecology remain poorly understood [37]. A large-scale isolation study of fission yeasts has demonstrated that *S. pombe*, *S. octosporus*, and *S. japonicus* occupy distinct habitats [38]. *S. pombe* and *S. octosporus* are primarily isolated from honey, and both species are also found in specific dried fruits that are probably visited by honeybees; *S. pombe* is additionally isolated from grape mash and fermented raw cacao beans. In contrast, *S. japonicus* is isolated mainly from forest materials, but also from fruits and fermented fruit products. Although *S. pombe* has been isolated from substrates associated with both temperate and tropical regions, the ecological distributions of other fission yeast species remain poorly understood. For example, *S. japonicus* has been frequently isolated in Japan [27], but whether this reflects a true climatic preference or a sampling bias is unclear. Future large-scale ecological surveys may reveal whether individual *Schizosaccharomyces* species occupy distinct climatic niches.

Although this study has revealed substantial diversity in spore shape and surface architecture among fission yeasts, the invaginated structures of the spore plasma membrane are conserved. Thus, fission yeasts seem to have diversified their life strategies primarily by modifications of spore wall structure rather than through changes in internal spore architecture. Because spores represent a major dispersal strategy in fungi, the acquisition of species-specific external spore structures is likely to have led to differences in interactions with vectors, stress tolerance, and surface hydrophobicity, thereby facilitating adaptation to distinct ecological niches.

## Materials & methods

### Yeast strains, media, and culture conditions

The yeast strains used in this study are listed in Table 1. Complete medium (YE) was used for vegetative growth; malt extract medium (ME) and synthetic sporulation media (MM-N and PMG) were used for mating and sporulation [39,40]. Solid media were prepared by adding 15 g/L of agar. Unless otherwise stated, yeast cells were incubated at 30 °C.

**Table 1:** Yeast strains used in this study.

Strains	NBRP ID^a^	Species	Source
**L968**	**FY7520**	***S. pombe***	**Leupold, 1948**
**yFS275/ATCC10660**	**FY16936**	***S. japonicus***	**Yukawa and Maki, 1931**
**yFS286/ATCC4206-U**	**FY16937**	***S. octosporus***	**Beijerinck, 1894**
**JB3/ATCC9987**	**FY32181**	***S. versatilis***	**Wickerham and Duprat, 1945**
**KYC800**	**BY19600**	***Saccharomyces cerevisiae***	**Fukunishi *et al.,* 2014**

^a^NBRP ID are from the National BioResource Project (NBRP; https://yeast.nig.ac.jp/yeast/).

### Quantification of mating rate

Cells grown overnight on YE agar medium were transferred to sporulation media and incubated for 3 days at 25 and 30 °C, or 5 days at 15 and 20 °C. Cells were counted using a DM500 microscope, and images were captured with an ICC50 wireless HD camera controlled by Leica AirLab software (Leica Microsystems, Tokyo, Japan). Cells were classified into four categories: vegetative cells (V), zygotes (Z), asci (A), and spores (S). The mating rate was calculated using the following equations [20]:

For *S. pombe*:


$$\begin{array}{c} \text{Mating rate }(\%)=\text{1}\text{0}\text{0}\times \frac{\text{2}Z+\text{2}A+S/\text{2}}{V+\text{2}Z+\text{2}A+S/\text{2}} \end{array}$$

For *S. japonicus, S. versatilis,* and *S. octosporus*:


$$\begin{array}{c} \text{Mating rate }(\%)=\text{1}\text{0}\text{0}\times \frac{\text{2}Z+\text{2}A+S/\text{4}}{V+\text{2}Z+\text{2}A+S/\text{4}} \end{array}$$

In all cases, three biological replicates were analyzed. For each replicate, 10 randomly selected digital images were acquired, and the mean ± SD was calculated.

### Optical microscopy

Living cells suspended in water were observed using a BX53 microscope (Olympus, Tokyo, Japan). Images were captured with a complementary metal–oxide–semiconductor (CMOS) camera (ORCA-Flash 4.0; Hamamatsu Photonics, Hamamatsu, Japan) controlled by HCImage Live software (Hamamatsu Photonics). Image processing was performed using ImageJ (National Institutes of Health, Bethesda, MD, USA).

### Spore shape analysis

Spore shape was evaluated by the AR, calculated with ImageJ. Bright-field images acquired with the BX53 microscope were processed using the *Find Edges* and *Threshold* commands. When necessary, the *Binary* command was also applied. The *Analyze Particles* command was used to measure the AR after selecting *Set Measurements > Shape Descriptors*. At least 200 spores were analyzed per sample, and the mean ± SD was calculated. In parallel, crescent-like spore shapes of *S. japonicus* and *S. versatilis* were evaluated by defining a crescent-like shape as “the ratio of the distances from the intersection of the long and short axes to each end of the short axis” (Fig 1D). To calculate this parameter, the long and short axes of each spore were drawn, and three key points were defined: both ends of the short axis and the intersection of the long and short axes. The (*X*, *Y*) coordinates of each point were obtained using the *Analyze Particles* command. The crescent-like shape was then calculated by the following equation:


$$\begin{array}{c} \text{crescent}-\text{like shape}=\frac{r_{\text{2}}}{r_{\text{1}}}=\sqrt{\frac{{\left(X_{\text{2}}-X_{\text{3}}\right)}^{\text{2}}+{\left(Y_{\text{2}}-Y_{\text{3}}\right)}^{\text{2}}}{{\left(X_{\text{1}}-X_{\text{3}}\right)}^{\text{2}}+{\left(Y_{\text{1}}-Y_{\text{3}}\right)}^{\text{2}}}} \end{array}$$

### Ultrathin-section electron microscopy

Ultrathin-section electron microscopy was performed by Tokai Electron Microscopy, Inc. (Nagoya, Japan) using a rapid freezing and freeze-substitution method. Sporulated cells cultured for more than 3 days were collected and washed twice with distilled water, then sandwiched between copper plates, and rapidly frozen in liquefied propane at −175°C. Frozen samples were treated with 2% glutaraldehyde and 1% tannic acid in ethanol containing 2% distilled water at −80 °C for 48 h. Subsequently, the temperature was increased gradually first to −20 °C in 3 h, and then to 4 °C in an additional 3 h.

Samples were dehydrated three times with absolute ethanol for 30 min each at room temperature and further incubated in absolute ethanol overnight. They were then treated twice with propylene oxide (PO) for 30 min and infiltrated with a 7:3 mixture of PO and resin (Quetol-651; Nisshin EM Co., Tokyo, Japan) for 1 h at room temperature. After evaporation of PO overnight at room temperature, samples were embedded in Quetol-651 resin at 60°C for 48 h. Ultrathin sections (80-nm thick) were prepared using an ultramicrotome (Ultracut UCT; Leica, Vienna, Austria) equipped with a diamond knife. Sections were mounted on copper grids, stained with 2% uranyl acetate for 15 min, followed by lead stain solution (Sigma-Aldrich Co.) for 3 min at room temperature. Observations were performed using a transmission electron microscope (JEM-1400 Plus; JEOL Ltd., Tokyo, Japan) operated at 100 kV, and images were captured with a CCD camera (EM-14830 RUBY2; JEOL Ltd.). A total of 11 *S. pombe*, 6 *S. octosporus*, 11 *S. japonicus*, and 21 *S. versatilis* spores were analyzed.

### Quick-freeze deep-etch replica electron microscopy

Quick-freeze deep-etch replica electron microscopy was performed as described previously [25]. Sporulated cells were prepared as described above and processed for replica preparation. Replicas were observed using a transmission electron microscope (JEM1010; JEOL Ltd.) operated at 80 kV. Images were captured with a FastScan-F214 (T) charge-coupled device (CCD) camera (TVIPS, Gauting, Germany). Due to the technical difficulty of replica preparation, the number of spores in which plasma membrane invaginations could be clearly evaluated was limited; nevertheless, invagination structures were successfully examined in 9 *S. pombe*, 9 *S. octosporus*, 2 *S. japonicus*, and 2 *S. versatilis* spores.

### Isolation of spores by density gradient centrifugation

Cells were sporulated for more than 5 days to allow spontaneous release of single spores. Spores were isolated using a linear 25%–55% Urografin (Bayer, Leverkusen, Germany) density-gradient centrifugation method, as described previously [41].

### Assessment of stress sensitivity

Stress sensitivity was assessed as described previously [12]. Isolated spores were exposed to either heat (55°C) or ethanol (30%) stress for 1, 2, or 3 h. After treatment, spores were plated on YE agar medium at appropriate dilutions to determine viability. Each experiment was performed in triplicate, and the mean ± SD was calculated.

## Supporting information

S1 Fig Phylogenetic tree of the genus *Schizosaccharomyces.* Modified from[1]. Divergence times between *S. cerevisiae* and the fission yeasts were taken from [2]. A mammalian phylogenetic tree is shown below the fission yeast tree to provide a reference for the evolutionary timescale and to compare the divergence of fission yeasts with that of mammals. Each divergence time in the mammalian phylogeny was based on [34–5]. MYA, million years ago.
(TIF)

S2 Fig Mating rate of fission yeasts on various sporulation media. (A) Effect of low temperature on mating rate in *S. pombe*, *S. octosporus*, *S. japonicus*, and *S. versatilis.* Each species was sporulated on PMG medium at various temperatures (15°C, 20°C, 25°C, 30°C). Triplicate samples were analyzed for each condition. Means and SD are shown. (B) *S. pombe*, *S. japonicus*, and *S. versatilis* were sporulated on other sporulation media (ME or MM-N agar medium) at various temperatures. Triplicate samples were analyzed for each condition. Means and SD are shown.
(TIF)

S3 Fig Detection of proteins associated with the outermost spore wall layer in fission yeasts. Proteins were extracted from the outermost spore wall layer and analyzed by SDS–PAGE as described previously [6]. The blue arrowhead indicates Isp3.
(TIF)

S4 Fig Comparison of the outermost spore layer between *S. cerevisiae* and fission yeasts. Autofluorescence of spores were observed by fluorescence microscopy as described previously [7]. Scale bars, 10 µm.
(TIF)

S5 FigAdhesion to pipette tips of the fission yeasts spores.(A) Representative images showing spore adhesion to the pipette tip. Pipette tips were dipped into a spore suspension (2.0 x 10^8^ spore/mL). The whitish area on the pipette tip (arrows) indicates spores adhered to the tip surface. (B) Quantification of spore adhered to the pipette tip. The mean gray values of the regions with and without adhered spores in (A) were measured using ImageJ. The relative gray value was calculated using the following equation: $Relativegrayvalue=\frac{meangrayvalueoftheregionwithadheredspores}{meangrayvalueoftheregionwithoutadheredspores}$(TIF)

S1 Table Spore wall formation related gene. In this study, CBS103 genome was used for searching *S. versatilis* gene. ^a^ the gene was conserved. ^b^ the gene was not conserved.
(PDF)

S2 Table Spore membrane formation related gene. In this study, CBS103 genome was used for searching *S. versatilis* gene. ^a^ the gene was conserved.
(PDF)

S6 Fig Original SDS-PAGE gel image corresponding to S3 Fig. (PDF)

S1 File Supplementary references. (DOCX)
