# Supplementary material for: Diversity of spore morphology among fission yeasts
Source: PLoS One. 2026 Aug 19;21(8):e0355697. doi: 10.1371/journal.pone.0355697 (PMC13489470; doi:10.1371/journal.pone.0355697)
Supplement: Supplementary file 1 [file pone.0355697.s006.pdf]

S1 Table. Spore wall formation related gene.

S. pombe	*S. octosporus*	*S. japonicucus*	*S. versatilis*	Product
agn2+	*SOCG_01070*	*SJAG_02718*	+ [a]	glucan endo-1,3-α-glucosidase
bgs2+	*SOCG_01669*	*SJAG_03587*	+	1,3-β-glucan synthase catalytic subunit
cda1+	*SOCG_03037*	*SJAG_03013*	+	chitin deacetylase
chs1+	*SOCG_00496*	*SJAG_04868*	+	chitin synthase Ⅰ
crp79+	*SOCG_01506*	*SJAG_03283*	+	Poly(A) binding protein
eng2+	*SOCG_02138*	*SJAG_01017*	+	cell wall and ascospore endo-1,3-β-glucanase
gas4+	*SOCG_00975*	*SJAG_04483*	+	spore wall 1,3-β-glucanosyltransferase
isp3+	-- [b]	-- [b]	-- [b]	spore wall structural constituent
meu10+	*SOCG_04001*	*SJAG_03483*	+	spore wall assembly GPI anchored protein
mok12+	*SOCG_00050*	*SJAG_03057*	+	spore wall-α-1,3-glucan synthase
mok13+	*SOCG_01285*	*SJAG_04184*	+	spore wall-α-1,3-glucan synthase
mok14+	*SOCG_03952*	*SJAG_04236*	+	spore wall-α-1,4-glucan synthase
rgf2+	*SOCG_01012*	*SJAG_04771*	+	RhoGEF for Rho1

In this study, CBS103 genome was used for searching *S. versatilis* gene.

[a] the gene was conserved. [b] the gene was not conserved.
