# Supplementary material for: Diversity of spore morphology among fission yeasts
Source: PLoS One. 2026 Aug 19;21(8):e0355697. doi: 10.1371/journal.pone.0355697 (PMC13489470; doi:10.1371/journal.pone.0355697)
Supplement: Supplementary file 2 [file pone.0355697.s007.pdf]

S2 Table. Spore membrane formation related gene.

S. pombe	*S. octosporus*	*S. japonicus*	*S. versatilis*	Product
meu14+	*SOCG_01485*	*SJAG_00405*	+ [a]	sporulation specific PIL domain protein
psy1+	*SOCG_04050*	*SJAG_03536*	+	plasma membrane and forespore membrane t-SNARE, syntaxin
spo3+	*SOCG_04388*	*SJAG_04922*	+	sporulation protein
spo9+	*SOCG_03581*	*SJAG_00580*	+	farnesyltranstransferase subunit
spo13+	*SOCG_05444*	*SJAG_02529*	+	sporulation specific RabGEF
spo14+	*SOCG_03615*	*SJAG_04615*	+	GEF for Sar1, WD repeat protein
spo15+	*SOCG_01937*	*SJAG_05309*	+	mitotic and meiotic spindle pole body protein
spo20+	*SOCG_01943*	*SJAG_03746*	+	phospholipid-intermembrane transfer protein

In this study, CBS103 genome was used for searching *S. versatilis* gene.

[a] the gene was conserved.
