# Supplementary material for: Diversity of spore morphology among fission yeasts
Source: PLoS One. 2026 Aug 19;21(8):e0355697. doi: 10.1371/journal.pone.0355697 (PMC13489470; doi:10.1371/journal.pone.0355697)
Supplement: Supplementary file 4 [file pone.0355697.s011.pdf]

PONE-D-26-13693R1. Sakaguchi et al. Diversity of Spore Morphology among Fission Yeasts

Authors:

There are two experimental and presentation issues that need to be address.

1. As Reviewer 1 points out, the evolutionary distance between the yeasts compared should be shown in an explanatory figure.

Following the reviewer's suggestion, we generated a phylogenetic tree and included it as S1 Fig. Although the reviewer suggested including comparisons with humans and mice, we limited the analysis to yeast species because the evolutionary distances between fission yeasts and mammals are extremely large, and the primary objective of this study is to compare spore morphology among fission yeast species.

Reviewer Response:

I think the comparison to mammalian evolution is an important point to be included, and the figure does not have to be a complete dendrogram with humans and fission yeasts. It is important because, especially with the rise conservative governments and the attitudes towards basic research compared to applied biomedical research, the profound nature of the Schizosaccharomyces species divergence will be overlooked if it is not driven home. I have multiple, personal examples of anthropocentric researchers claiming that a protein is conserved across mammals is important when that conservation is insignificant. This issue will persist and grow long after the senior author of this manuscript, this reviewer and the editor have been forced out of science. This figure has the opportunity to make a slide that can be used by many fission yeast researchers in the future to show how useful these comparisons are for protein and cellular evolution. I will add that my colleagues in the budding yeast community had to make similar arguments when discussing the evolutionary divergence of Saccharomyces species decades ago. The divergence of Schizosaccharomyces species is even more ancient, so the evolutionary comparisons are more meaningful.

I am providing a modified version of Figure S1 in this pdf to explain what I mean, including references not intended for a final figure. Briefly, small labels show when humans diverged from other mammals at the bottom of the main fission yeast dendrogram, so one can use this as a guide for the evolutionary time scale. (I will leave it to future users to add little silhouettes or illustrations for these evolutionary landmarks.) The bottom of the figure includes a time scale I made to place the tick marks and the references use to give the divergence times from present day (humans-cows 85 Mya, humans-mice 92 Mya, humans-kangaroos(marsupials) 148 Mya, humans-platypus(monotremes) 166 Mya, first mammalian fossil 225 Mya, first reptile 318 Mya) so the authors can judge the figure before making their own version.

A remaining open question is whether early pre-humans made pombe beer or used the other yeast.

Fig. S1 suggestion:

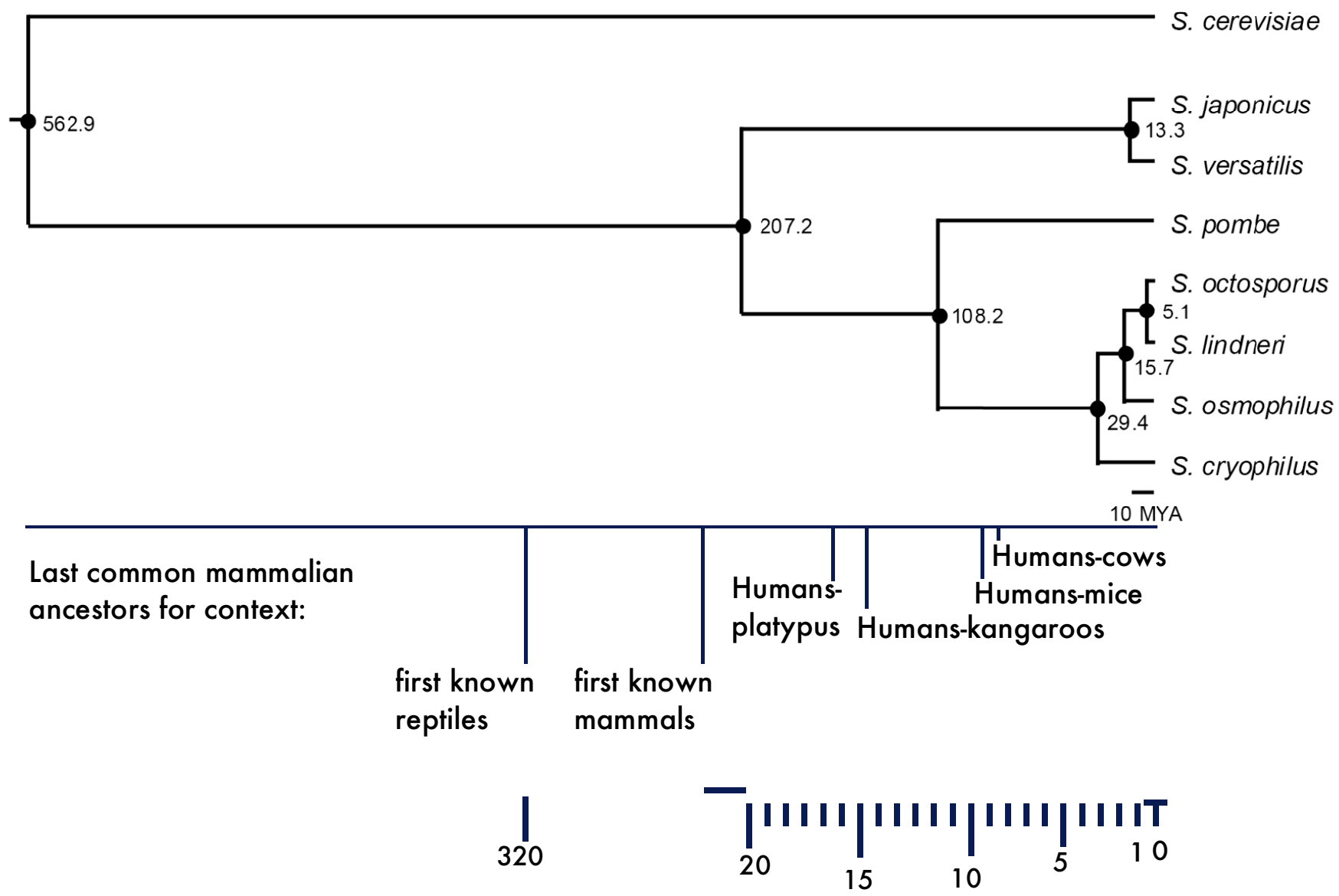

reptiles emerge PMID: 31900445 318 Mya
first known mammals: PMID: 36065514 225 Mya
Mammalian divergence points: PMID: 17392779

Authors:

2. The completeness of the study would be enhanced by a comparison of some of the other mutations that effect spore morphology as Reviewer 1 points out.

Reviewer Response:

The authors response satisfies my concern.

Issues and typographical errors:

1. Lines 238-239 vs. Fig. S2A: “that of *S. octosporus* increased with decreasing temperature, reaching ~70% at both 20°C and 15°C. *S. versatilis* exhibited the highest mating rate (~70%) at 30°C, with a gradual decline at lower temperatures”

I think that S. octosporus (So) and S. versatilis (Sv) are switched in this sentence, or the Fig 2A bar graph is mislabeled. In my S2_fig.tif file downloaded with the revised manuscript, the frequency of So sporulation is lowest at 15 C and highest at 30C. The Sv sporulation frequency is highest at 15 C while 25 and 20 C are similar.
The rest of the paragraph should be checked for accuracy.

2. Lines 306-307: “these fission yeast spores did not fluoresce under ultraviolet light, a characteristic feature of the dityrosine layer (S4 Fig).”

I believe that Fig. S3 is what the authors meant.

3. Line 340: “included within the limited set of 100–400 non-conserved genes in fission yeasts”

The authors should provide a reference or references for where these numbers come from. I am guessing that these are summaries from the genome sequences of the different species compared with S. pombe but it would be helpful to others to give the basis for this statement.

4. Lines 602-604 and Fig. S5 legend: “The mean gray values of the regions indicated by arrows”

It took me some time to interpret this figure. I believe that the “gray area” in the pipet tip is spores that adhere to the tip, showing the hydrophobic interaction.

Please add a sentence to the legend for Fig. S5A explaining that the gray area are spores that remain adhered to the pipet tip.

The Methods section does not describe how this test was done. A brief explanation could be in either the Methods or the Fig. S5 legend.
