# Supplementary material for: Diversity of spore morphology among fission yeasts
Source: PLoS One. 2026 Aug 19;21(8):e0355697. doi: 10.1371/journal.pone.0355697 (PMC13489470; doi:10.1371/journal.pone.0355697)
Supplement: Supplementary file 5 [file pone.0355697.s009.docx]

**References**

1. Brysch-Herzberg M, Jia GS, Sipiczki M, Seidel M, Zhang WC, Du LL. Reinstatement of the fission yeast species *Schizosaccharomyces versatilis* Wickerham et Duprat, a sibling species of Schizosaccharomyces japonicus. Yeast. 2024;41(3):108–27.

2. Shen XX, Steenwyk JL, LaBella AL, Opulente DA, Zhou X, Kominek J, *et al*.Genome-scale phylogeny and contrasting modes of genome evolution in the fungal phylum Ascomycota. Sci Adv. 2020;6(45).

3. Ford DP, Benson RBJ. The phylogeny of early amniotes and the affinities of Parareptilia and Varanopidae. Nat Ecol Evol. 2020;4(1):57–65.

4. Cabreira SF, Schultz CL, da Silva LR, Lora LHP, Pakulski C, do Rego RCB, *et al*. Diphyodont tooth replacement of *Brasilodon*-A Late Triassic eucynodont that challenges the time of origin of mammals. J Anat. 2022;241(6):1424–40.

5. Bininda-Emonds OR, Cardillo M, Jones KE, MacPhee RD, Beck RM, Grenyer R, *et al*. The delayed rise of present-day mammals. Nature. 2007;446(7135):507–12.

6. Fukunishi K, Miyakubi K, Hatanaka M, Otsuru N, Hirata A, Shimoda C, *et al*. The fission yeast spore is coated by a proteinaceous surface layer comprising mainly Isp3. Mol Biol Cell. 2014;25(10):1549–59.

7. Briza P, Winkler G, Kalchhauser H, Breitenbach M. Dityrosine is a prominent component of the yeast ascospore wall. A proof of its structure. J Biol Chem. 1986;261(9):4288–94.
