# Supplementary material for: Diversity of spore morphology among fission yeasts
Source: PLoS One. 2026 Aug 19;21(8):e0355697. doi: 10.1371/journal.pone.0355697 (PMC13489470; doi:10.1371/journal.pone.0355697)
Supplement: Supplementary file 6 [file pone.0355697.s012.docx]

June 10, 2026

Dear Editor,

We greatly appreciate the constructive comments of the reviewers and the decision of the editor. In accordance with these comments, we have revised our manuscript appropriately (Manuscript : *PONE-D-26-13693*)

Below we give an itemized account of our revisions in response to the reviewers’ comments.

==============================
**There are two experimental and presentation issues that need to be address.**

**1. As Reviewer 1 points out, the evolutionary distance between the yeasts compared should be shown in an explanatory figure.**

Following the reviewer's suggestion, we generated a phylogenetic tree and included it as S1 Fig. Although the reviewer suggested including comparisons with humans and mice, we limited the analysis to yeast species because the evolutionary distances between fission yeasts and mammals are extremely large, and the primary objective of this study is to compare spore morphology among fission yeast species.

**2. The completeness of the study would be enhanced by a comparison of some of the other mutations that effect spore morphology as Reviewer 1 points out.**

In response to the reviewer’s suggestion, we have added a new supplementary Table (S2 Table) showing the presence or absence of homologs of genes involved in spore membrane formation, including *spo3^+^* and *psy1^+^*, across the fission yeast species examined in this study. We also added a corresponding description to the revised manuscript (Lines 287–288, 337).

**In addition, Reviewer 1 indicated that some conclusions are overstated. Please rewrite each of these sections to accommodate these concerns.**

We agree with the reviewer's comments and have revised the relevant statements throughout the manuscript to use more appropriate and cautious wording.

==============================
**Journal Requirements:**
We have addressed all editorial and administrative requirements requested by the journal, including funding information, data availability, code sharing, figure copyright, and reference list verification.

**1. PLOS ONE formatting requirements**
The manuscript and associated files have been revised to comply with PLOS ONE formatting and file-naming requirements.

**2. Code sharing**
We confirm that this study did not rely on any author-generated code for data analysis or generation of the reported findings. Therefore, the PLOS ONE code sharing policy is not applicable to this manuscript.

**3. Funding information**
We checked the grant information and corrected any inconsistencies between the Funding Information and Financial Disclosure sections.

**4. Funding statement and role of funders**
The Funding Statement has been revised accordingly. The funders had no role in study design, data collection and analysis, decision to publish, or preparation of the manuscript.

**5. Finance disclosure**
The funders had no role in study design, data collection and analysis, decision to publish, or preparation of the manuscript.

**6. Data availability**
We confirm that all relevant data will be made publicly available upon publication in accordance with the PLOS ONE data sharing policy.

**7. “Data not shown”**
We have removed all instances of the phrase “data not shown” from the manuscript. The corresponding data have been included in the Supplementary Materials, and the relevant sections of the manuscript have been revised accordingly.

**8. Copyrighted figures**
We have reviewed all figures and confirm that they are original works created by the authors and do not contain third-party copyrighted material.

**9. Suggested references**
We evaluated the suggested references and determined that no additional citations were required.

**10. Reference list**
We reviewed the reference list and corrected it where necessary. No retracted articles are cited in the manuscript.

Reviewers' comments:
Reviewer's Responses to Questions
**Comments to the Author**
 **Reviewer #1: This is a descriptive comparative study of the spore morphology of the four major fission yeast species. The main and interesting findings are that the morphologies are distinct, and correlate with different levels of resistance of the spores to environmental stressors. An interesting minor finding is that mating efficiency in octosporus is extremely temperature sensitive. I have no major concerns with this, and only a few minor comments.

The authors state that the protein Isp3 which coats the outside of S. pombe spores is not found in octosporus but is it found in japonicus?**

We thank the reviewer for this comment. We have clarified that neither *S. octosporus* nor *S. japonicus* possesses a close homolog of Isp3 and revised the relevant text accordingly. The corresponding changes have been made in the revised manuscript (Lines 287–295).

**Line 336: RNAi is known to be required for S. pombe silencing at centromeres (as written the statement implies it is not).**

We agree with the reviewer. Our original statement was based on a misinterpretation of the cited reference. Therefore, we have removed the relevant text from the revised manuscript (Line 358).

**In the discussion, the authors might want to speculate a bit more about the habitats of these species. pombe at least is widely dispersed in temperate as well as tropical climates. Are the others more temperate/northerly?**

We thank the reviewer for this interesting suggestion. At present, there are limited data linking the distribution of fission yeast species other than *S. pombe* to specific climate zones. To our knowledge, previous studies have not reported whether *S. japonicus* or *S. octosporus* are preferentially associated with temperate, tropical, or northern environments. However, *S. japonicus* has been frequently isolated in Japan (Seike *et al.,* 2021), suggesting that it is well established in at least some temperate regions.
 In addition, Brysch-Herzberg *et al.,* 2022 emphasized the importance of comprehensive isolation studies to identify the natural substrates from which each *Schizosaccharomyces* species can be most effectively recovered. Because the currently known isolation sources are geographically and environmentally biased, it remains difficult to draw firm conclusions regarding the climatic preferences of individual fission yeast species other than *S. pombe*. Future ecological surveys and broader sampling efforts may help clarify whether different *Schizosaccharomyces* species are associated with particular climate zones or habitats.
The corresponding changes have been made in the revised manuscript (Lines 370-375).

**One feature not discussed much is that pombe is a 4-spored species but all the others produce 8 spores, presumably due to an extra mitosis. Could this be related to the spore morphology? Perhaps some speculation....**

Whereas *S. pombe* typically produces four spores per ascus, *S. octosporus*, *S. japonicus*, and *S. versatilis* usually produce eight spores. However, asci containing only four spores are also occasionally observed in these species. Examination of these spores revealed that none exhibited the bumpy surface characteristic of *S. pombe*. These observations suggest that spore morphology is not related to the number of spores formed within an ascus.

The corresponding changes have been made in the revised manuscript (Lines 297–302).

**Similarly, is there anything known about the rate of germination? that is, how fast after re-feeding do these spores enter vegetative growth?**

The germination frequencies were similar among *S. pombe* (>90%), *S. japonicus* (88%), and *S. octosporus* (83%) (n > 150), indicating no substantial differences among these species. In contrast, the germination frequency of *S. versatilis* was approximately 20% (n > 150). However, these experiments were performed on YE medium, which may not represent the optimal growth conditions for *S. versatilis*. While we assessed germination frequencies, we did not measure germination kinetics. To our knowledge, no studies have systematically compared germination rates among fission yeast species. The corresponding changes have been made in the revised manuscript (Lines 303–307).

**Reviewer #2: Review of PONE-26-13693, Sakaguchi et al.,

The manuscript examines the spores formed by four different, evolutionarily diverse Schizosaccharomyces species using light and electron microscopy and stress resistance. The work fills a gap in the characterization of fission yeasts, as the species examined are thought to have diverged about 200 million years ago (Mya) and the findings are quite diverse from the best studied system, S. pombe.

The manuscript is reasonably complete, but contains mistakes, presents some correlations too strongly and equivocates on more recent data shown by multiple labs. These issues are enumerated below and need to be addressed.**

**Major:

1) Line 67 and a new explanatory figure. The manuscript compares S. pombe, S. octosporus, S. japonicus and S. versatilis, but the evolutionary distance between these yeasts is not well presented. This reviewer suggests a figure with an evolutionary tree showing divergence of these four fission yeasts, similar to Fig. 3 in Brysch-Hershberg et al. Yeast. 2024;41:108–127. I also strongly suggest that below the yeast dendrogram and comparative dendrogram for humans and rodents such as in COMMUNICATIONS BIOLOGY | (2022) 5:986. Inclusion of an aligned panel of mammalian evolution would show that the authors are comparing yeast that diverged long before humans and rodents, and would drive home how different the yeasts are, as many labs studying mammalian systems think all yeasts are the same.**

We thank the reviewer for this helpful suggestion. To clarify the evolutionary relationships among the fission yeasts examined in this study, we have added a new explanatory figure showing a phylogenetic tree of *S. pombe, S. octosporus, S. japonicus*, and *S. versatilis*, based on the recent classification and phylogenetic analysis reported by Brysch-Herzberg *et al,* 2024. This figure is now included as S1 Fig, and the relevant description has been added to the revised manuscript. (Line 62).

We agree that comparison with mammalian divergence would be useful for emphasizing the large evolutionary distances among fission yeasts. However, because the focus of the present study is the diversity of spore morphology within the genus *Schizosaccharomyces*, we have limited the new figure to the yeast species analyzed in this study. We have instead clarified in the text that these fission yeasts are evolutionarily distant species, so that readers can better appreciate that they should not be regarded as closely related variants of the same yeast model.

**2) Lines 60, 117 and 271 and S. japonicus versus S. versatilis as different species. The authors equivocate as to whether the two yeasts are different species or varieties of the same species, even though they cite recent papers with whole genome sequencing and genetic data that indicates that the two yeasts are different species. Why do the authors say “suggested that…represents two different species”? Why do the authors equivocate? They should give some reason why they doubt the species reclassification.**

We agree with the reviewer's comment. In accordance with recent genomic studies supporting the classification of *S. japonicus* and *S. versatilis* as distinct species, we have revised the text to avoid any ambiguity. The corresponding revisions are provided in the revised manuscript (Lines 117–118, 276-278).

**3) Lines 111-182, 301-303, 353: plasma membrane structures are “highly conserved”. I take issue with the term “highly” because the figure presents plasma membranes with parallel invaginations but the structures presented look very different between species. While Saccharomyces cerevisiae has invaginations that are not parallel, the authors appear to be overstating their observation.**

We agree with the reviewer's comment that the term "highly conserved" overstates our observations. To more accurately describe the results, we have revised the text to state that "the presence of parallel invaginations is conserved" among these species. The corresponding changes have been made in the revised manuscript (Lines 30, 159-160, 165, 167-168, 320-321, 378).

**4) In the observation of the plasma membranes by QFDE-EM, the authors never state how many cells were observed for each species where invaginations can be judged. The authors need to present these numbers in the Results or Methods.**

We agree that the number of spores examined by QFDE-EM should be stated. We have now added the number of spores analyzed for each species in the Materials and Methods section. By QFDE-EM, although the number of replicas obtained was limited because of the technical difficulty of replica preparation, we examined 9 spores of *S. pombe*, 9 spores of *S. octosporus*, 2 spores of *S. japonicus*, and 2 spores of *S. versatilis*. In addition, similar membrane invaginations were independently confirmed by transmission electron microscopy of ultrathin sections, in which 11 *S. pombe*, 6 *S. octosporus*, 11 *S. japonicus*, and 21 *S. versatilis* spores were analyzed (Fig 5). Together, these observations support the presence of membrane invaginations in all four species examined. The corresponding changes have been made in the revised manuscript (Lines 451-452, 459-463).

**5) Line 205 – probably corresponds to the fibrillar structure. It is hard to know what this statement means and judge if it is true. The electron dense surface appears to be the surface of the spore, supported by the underlying electron transparent layer. The connection between the bumpy spore surface and the fibrillar adjective is not clear (I was expecting lines or cables or fibrils). The statement should either be explained or deleted.**

We agree that the connection between the electron-dense outermost layer and the fibrillar surface structures was not sufficiently explained. In our previous study (Fukunishi *et al.,* 2014), these two structures were shown to correspond to the same outermost spore wall layer. We have therefore revised the text to explicitly describe this relationship and added a reference to the corresponding QFDE-EM image (Fig 2A, lower panel). The corresponding changes have been made in the revised manuscript (Lines 207–209).

**6) Lines 249-251 – The conclusion suggesting a link between stress resistance and spore wall differences is a correlation that has not been tested, and is overstated. Line 251 could state that spores are “structurally distinct and correlates with stress resistance.”**

We agree with the reviewer that the previous statement was overstated. We have revised the text to clarify that the observed relationship between spore wall structure and stress resistance is a correlation. The corresponding changes have been made in the revised manuscript (Lines 254–255).

**7) Lines 288-289 – The discussion of the lack of a protein layer for some spore types is based on data not shown and the absence of an electron dense layer, which is not convincing as presented. The protein gels should be presented in the Supplemental Material and the conclusion about a protein layer needs better justification. The lack of staining in the EM is not sufficient to say no protein layer exists.**

We agree with the reviewer’s comment. We have now included the SDS–PAGE data in the Supplemental Material (S3 Fig). Extraction of the outermost layer of *S. octosporus* spores yielded two protein bands on SDS–PAGE; however, both bands were weaker and differed in molecular size from the S. pombe Isp3 protein, indicating that *S. octosporus* spores possess a proteinaceous surface component distinct from the Isp3 layer of *S. pombe*. In contrast, no distinctive protein bands were detected in *S. japonicus* or *S. versatilis* spores subjected to the same extraction procedure (S3 Fig). Based on these results, we have revised our interpretation and no longer conclude that these species completely lack an outer protein layer. Instead, we conclude that our data do not support the presence of a prominent proteinaceous outer spore wall layer comparable to the Isp3 layer of *S. pombe*. The corresponding revisions have been incorporated into the revised manuscript (Lines 285–295).


**8) Line 326 – There are a number of genes in spore wall formation, some like spo3 and psy1 studied by the Nakamura lab, that may play a role in the different spore ultra structures. A complete presentation would include whether the homologs of the genes in this process are present across fission yeast evolution.**

In response to the reviewer’s suggestion, we have added a new supplementary Table (S2 Table) showing the presence or absence of homologs of genes involved in forespore membrane formation, including *spo3^+^* and *psy1^+^*, across the fission yeast species examined in this study. We also added a corresponding description to the revised manuscript (Lines 287–288, 337).


**Minor.**
We thank the reviewer for this suggestion and have revised the manuscript accordingly.

In summary, we have carefully considered all of the reviewers’ comments and revised the manuscript accordingly. We hope that our manuscript is now acceptable for publication in *PLOS ONE*.

Sincerely yours,

Taro Nakamura
