# Supplementary material for: Diversity of spore morphology among fission yeasts
Source: PLoS One. 2026 Aug 19;21(8):e0355697. doi: 10.1371/journal.pone.0355697 (PMC13489470; doi:10.1371/journal.pone.0355697)
Supplement: Peer review history

# Peer review: Decision Letter 0

**DOI:** 10.1371/journal.pone.0355697.r001

**Authors:** Arthur Lustig

28 Apr 2026

Dear Dr. Nakamura,

==============================

**There are two experimental and presentation issues that need to be address** **In addition, Reviewer 1 indicated that some conclusions are overstated. Please rewrite each of these sections to accommodate these concerns.**

==============================

Please submit your revised manuscript by Jun 12 2026 11:59PM. If you will need more time than this to complete your revisions, please reply to this message or contact the journal office at plosone@plos.org. When you're ready to submit your revision, log on to https://www.editorialmanager.com/pone/ and select the 'Submissions Needing Revision' folder to locate your manuscript file.

- A letter that responds to each point raised by the academic editor and reviewer(s). You should upload this letter as a separate file labeled 'Response to Reviewers'.
- A marked-up copy of your manuscript that highlights changes made to the original version. You should upload this as a separate file labeled 'Revised Manuscript with Track Changes'.
- An unmarked version of your revised paper without tracked changes. You should upload this as a separate file labeled 'Manuscript'.

If you would like to make changes to your financial disclosure, please include your updated statement in your cover letter. Guidelines for resubmitting your figure files are available below the reviewer comments at the end of this letter.

If applicable, we recommend that you deposit your laboratory protocols in protocols.io to enhance the reproducibility of your results. Protocols.io assigns your protocol its own identifier (DOI) so that it can be cited independently in the future. For instructions see: https://journals.plos.org/plosone/s/submission-guidelines#loc-laboratory-protocols. Additionally, PLOS ONE offers an option for publishing peer-reviewed Lab Protocol articles, which describe protocols hosted on protocols.io. Read more information on sharing protocols at https://plos.org/protocols?utm_medium=editorial-email&utm_source=authorletters&utm_campaign=protocols.

As the corresponding author, your ORCID iD is verified in the submission system and will appear in the published article. PLOS supports the use of ORCID, and we encourage all coauthors to register for an ORCID iD and use it as well. Please encourage your coauthors to verify their ORCID iD within the submission system before final acceptance, as unverified ORCID iDs will not appear in the published article. *Only*  the individual author can complete the verification step; PLOS staff *cannot*  verify ORCID iDs on behalf of authors.

We look forward to receiving your revised manuscript.

Kind regards,

Arthur J. Lustig, PhD

Academic Editor

PLOS One

**Journal Requirements:**

1. When submitting your revision, we need you to address these additional requirements.

Please ensure that your manuscript meets PLOS ONE's style requirements, including those for file naming. The PLOS ONE style templates can be found at

https://journals.plos.org/plosone/s/file?id=wjVg/PLOSOne_formatting_sample_main_body.pdf and

https://journals.plos.org/plosone/s/file?id=ba62/PLOSOne_formatting_sample_title_authors_affiliations.pdf

2. Please note that PLOS One has specific guidelines on code sharing for submissions in which author-generated code underpins the findings in the manuscript. In these cases, we expect all author-generated code to be made available without restrictions upon publication of the work. Please review our guidelines at https://journals.plos.org/plosone/s/materials-and-software-sharing#loc-sharing-code and ensure that your code is shared in a way that follows best practice and facilitates reproducibility and reuse.

3. We note that the grant information you provided in the ‘Funding Information’ and ‘Financial Disclosure’ sections do not match.

When you resubmit, please ensure that you provide the correct grant numbers for the awards you received for your study in the ‘Funding Information’ section.

4. Thank you for stating in your Funding Statement:

This work was supported by Japan Society for the Promotion of Science (JSPS) KAKENHI (18K06225, 21K06176, and 24K09457), Ohsumi Frontier Science Foundation to T.N., by a JST CREST grant (JPMJCR19S5) and Osaka City University (OCU) Strategic Research Grant 2018 for top priority researches to M.M. and JST SPRING, Grant Number JPMJSP2139 to T.S.

Please provide an amended statement that declares *all* the funding or sources of support (whether external or internal to your organization) received during this study, as detailed online in our guide for authors at http://journals.plos.org/plosone/s/submit-now.  Please also include the statement “There was no additional external funding received for this study.” in your updated Funding Statement.

Please include your amended Funding Statement within your cover letter. We will change the online submission form on your behalf.

5. Thank you for stating the following financial disclosure:

This work was supported by Japan Society for the Promotion of Science (JSPS) KAKENHI (18K06225, 21K06176, and 24K09457), Ohsumi Frontier Science Foundation to T.N., by a JST CREST grant (JPMJCR19S5) and Osaka City University (OCU) Strategic Research Grant 2018 for top priority researches to M.M. and JST SPRING, Grant Number JPMJSP2139 to T.S.

Please state what role the funders took in the study.  If the funders had no role, please state: "The funders had no role in study design, data collection and analysis, decision to publish, or preparation of the manuscript."

If this statement is not correct you must amend it as needed.

Please include this amended Role of Funder statement in your cover letter; we will change the online submission form on your behalf.

6. When completing the data availability statement of the submission form, you indicated that you will make your data available on acceptance. We strongly recommend all authors decide on a data sharing plan before acceptance, as the process can be lengthy and hold up publication timelines. Please note that, though access restrictions are acceptable now, your entire data will need to be made freely accessible if your manuscript is accepted for publication. This policy applies to all data except where public deposition would breach compliance with the protocol approved by your research ethics board. If you are unable to adhere to our open data policy, please kindly revise your statement to explain your reasoning and we will seek the editor's input on an exemption. Please be assured that, once you have provided your new statement, the assessment of your exemption will not hold up the peer review process.

7. We note that you have included the phrase “data not shown” in your manuscript. Unfortunately, this does not meet our data sharing requirements. PLOS does not permit references to inaccessible data. We require that authors provide all relevant data within the paper, Supporting Information files, or in an acceptable, public repository. Please add a citation to support this phrase or upload the data that corresponds with these findings to a stable repository (such as Figshare or Dryad) and provide and URLs, DOIs, or accession numbers that may be used to access these data. Or, if the data are not a core part of the research being presented in your study, we ask that you remove the phrase that refers to these data.

8. We note that Figures 1, 2, 3, 4, 5 and S1 in your submission contain copyrighted images. All PLOS content is published under the Creative Commons Attribution License (CC BY 4.0), which means that the manuscript, images, and Supporting Information files will be freely available online, and any third party is permitted to access, download, copy, distribute, and use these materials in any way, even commercially, with proper attribution. For more information, see our copyright guidelines: http://journals.plos.org/plosone/s/licenses-and-copyright.

We require you to either present written permission from the copyright holder to publish these figures specifically under the CC BY 4.0 license, or remove the figures from your submission:

a. You may seek permission from the original copyright holder of Figures 1, 2, 3, 4, 5 and S1 to publish the content specifically under the CC BY 4.0 license.

We recommend that you contact the original copyright holder with the Content Permission Form (http://journals.plos.org/plosone/s/file?id=7c09/content-permission-form.pdf) and the following text:

“I request permission for the open-access journal PLOS ONE to publish XXX under the Creative Commons Attribution License (CCAL) CC BY 4.0 (http://creativecommons.org/licenses/by/4.0/). Please be aware that this license allows unrestricted use and distribution, even commercially, by third parties. Please reply and provide explicit written permission to publish XXX under a CC BY license and complete the attached form.”

Please upload the completed Content Permission Form or other proof of granted permissions as an "Other" file with your submission.

In the figure caption of the copyrighted figure, please include the following text: “Reprinted from [ref] under a CC BY license, with permission from [name of publisher], original copyright [original copyright year].”

b. If you are unable to obtain permission from the original copyright holder to publish these figures under the CC BY 4.0 license or if the copyright holder’s requirements are incompatible with the CC BY 4.0 license, please either i) remove the figure or ii) supply a replacement figure that complies with the CC BY 4.0 license. Please check copyright information on all replacement figures and update the figure caption with source information. If applicable, please specify in the figure caption text when a figure is similar but not identical to the original image and is therefore for illustrative purposes only.

9. If the reviewer comments include a recommendation to cite specific previously published works, please review and evaluate these publications to determine whether they are relevant and should be cited. There is no requirement to cite these works unless the editor has indicated otherwise. 

10. Please review your reference list to ensure that it is complete and correct. If you have cited papers that have been retracted, please include the rationale for doing so in the manuscript text, or remove these references and replace them with relevant current references. Any changes to the reference list should be mentioned in the rebuttal letter that accompanies your revised manuscript. If you need to cite a retracted article, indicate the article’s retracted status in the References list and also include a citation and full reference for the retraction notice.

[Note: HTML markup is below. Please do not edit.]

Reviewers' comments:

**Comments to the Author**

1. Is the manuscript technically sound, and do the data support the conclusions?

Reviewer #1: Yes

Reviewer #2: Partly

**********

2. Has the statistical analysis been performed appropriately and rigorously?

Reviewer #1: Yes

Reviewer #2: No

**********

3. Have the authors made all data underlying the findings in their manuscript fully available?

The PLOS Data policy

Reviewer #1: Yes

Reviewer #2: Yes

**********

4. Is the manuscript presented in an intelligible fashion and written in standard English?

Reviewer #1: Yes

Reviewer #2: Yes

**********

**Reviewer #1:** This is a descriptive comparative study of the spore morphology of the four major fission yeast species. The main and interesting findings are that the morphologies are distinct, and correlate with different levels of resistance of the spores to environmental stressors. An interesting minor finding is that mating efficiency in octosporus is extremely temperature sensitive. I have no major concerns with this, and only a few minor comments.

The authors state that the protein Isp3 which coats the outside of S. pombe spores is not foud in octosporus but is it found in japonicus?

Line 336: RNAi is known to be required for S. pombe silencing at centromeres (as written the statement implies it is not).

In the discussion, the authors might want to speculate a bit more about the habitats of these species. pombe at least is widely dispersed in temperate as well as tropical climates. Are the others more temperate/northerly?

One feature not discussed much is that pombe is a 4-spored species but all the others produce 8 spores, presumably due to an extra mitosis. Could this be related to the spore morphology? Perhaps some speculation....

Similarly, is there anything known about the rate of germination? that is, how fast after re-feeding do these spores enter vegetative growth?

**Reviewer #2:**  Review of PONE-26-13693, Sakaguchi et al.,

The manuscript examines the spores formed by four different, evolutionarily diverse Schizosaccharomyces species using light and electron microscopy and stress resistance. The work fills a gap in the characterization of fission yeasts, as the species examined are thought to have diverged about 200 million years ago (Mya) and the findings are quite diverse from the best studied system, S. pombe.

The manuscript is reasonably complete, but contains mistakes, presents some correlations too strongly and equivocates on more recent data shown by multiple labs. These issues are enumerated below and need to be addressed.

Major:

1) Line 67 and a new explanatory figure. The manuscript compares S. pombe, S. octosporus, S. japonicus and S. versatilis, but the evolutionary distance between these yeasts is not well presented. This reviewer suggests a figure with an evolutionary tree showing divergence of these four fission yeasts, similar to Fig. 3 in Brysch-Hershberg et al. Yeast. 2024;41:108–127. I also strongly suggest that below the yeast dendrogram and comparative dendrogram for humans and rodents such as in COMMUNICATIONS BIOLOGY | (2022) 5:986. Inclusion of an aligned panel of mammalian evolution would show that the authors are comparing yeast that diverged long before humans and rodents, and would drive home how different the yeasts are, as many labs studying mammalian systems think all yeasts are the same.

2) Lines 60, 117 and 271 and S. japonicus versus S. versatilis as different species. The authors equivocate as to whether the two yeasts are different species or varieties of the same species, even though they cite recent papers with whole genome sequencing and genetic data that indicates that the two yeasts are different species. Why do the authors say “suggested that…represents two different species”? Why do the authors equivocate? They should give some reason why they doubt the species reclassification.

3) Lines 111-182, 301-303, 353: plasma membrane structures are “highly conserved”. I take issue with the term “highly” because the figure presents plasma membranes with parallel invaginations but the structures presented look very different between species. While Saccharomyces cerevisiae has invaginations that are not parallel, the authors appear to be overstating their observation.

4) In the observation of the plasma membranes by QFDE-EM, the authors never state how many cells were observed for each species where invaginations can be judged. The authors need to present these numbers in the Results or Methods.

5) Line 205 – probably corresponds to the fibrillar structure. It is hard to know what this statement means and judge if it is true. The electron dense surface appears to be the surface of the spore, supported by the underlying electron transparent layer. The connection between the bumpy spore surface and the fibrillar adjective is not clear (I was expecting lines or cables or fibrils). The statement should either be explained or deleted.

6) Lines 249-251 – The conclusion suggesting a link between stress resistance and spore wall differences is a correlation that has not been tested, and is overstated. Line 251 could state that spores are “structurally distinct and correlates with stress resistance.”

7) Lines 288-289 – The discussion of the lack of a protein layer for some spore types is based on data not shown and the absence of an electron dense layer, which is not convincing as presented. The protein gels should be presented in the Supplemental Material and the conclusion about a protein layer needs better justification. The lack of staining in the EM is not sufficient to say no protein layer exists.

8) Line 326 – There are a number of genes in spore wall formation, some like spo3 and psy1 studied by the Nakamura lab, that may play a role in the different spore ultra structures. A complete presentation would include whether the homologs of the genes in this process are present across fission yeast evolution.

Minor.

9) Line 56 – A reference is needed at the end of the line.

10) Fig. 1 – I downloaded the high resolution pictures they are still fuzzy, as can be seen in the lack of sharpness in the arrowheads. Be sure the best resolution figure possible is uploaded.

11) Lines 236-237 – the descriptions of the S. versatilis and S. octosporus spores are reversed from the data in Fig. 6 and need to be corrected.

12) Fig. S1 and Fig. S2 were switched in the uploading to the PLOS server, and need to be uploaded correctly in the revised version.

13) Table S1 – a superscript “b” is in the footnote but I did not see a superscript b in the body of the table.

**********

what does this mean?). If published, this will include your full peer review and any attached files.

If you choose “no”, your identity will remain anonymous but your review may still be made public.

**Do you want your identity to be public for this peer review?**  For information about this choice, including consent withdrawal, please see our Privacy Policy

Reviewer #1: No

Reviewer #2: No

**********

[NOTE: If reviewer comments were submitted as an attachment file, they will be attached to this email and accessible via the submission site. Please log into your account, locate the manuscript record, and check for the action link "View Attachments". If this link does not appear, there are no attachment files.]

To ensure your figures meet our technical requirements, please review our figure guidelines: https://journals.plos.org/plosone/s/figures

You may also use PLOS’s free figure tool, NAAS, to help you prepare publication quality figures: https://journals.plos.org/plosone/s/figures#loc-tools-for-figure-preparation.

NAAS will assess whether your figures meet our technical requirements by comparing each figure against our figure specifications.

# Author comment: Author response to Decision Letter 1

**DOI:** 10.1371/journal.pone.0355697.r002

10 Jun 2026

Dear Editor,

We greatly appreciate the constructive comments of the reviewers and the decision of the editor. In accordance with these comments, we have revised our manuscript accordingly (Manuscript ID: PONE-D-26-13693)

Below we give an itemized account of our revisions in response to the reviewers’ comments.

==============================

There are two experimental and presentation issues that need to be address.

1. As Reviewer 1 points out, the evolutionary distance between the yeasts compared should be shown in an explanatory figure.

Following the reviewer's suggestion, we generated a phylogenetic tree and included it as S1 Fig. Although the reviewer suggested including comparisons with humans and mice, we limited the analysis to yeast species because the evolutionary distances between fission yeasts and mammals are extremely large, and the primary objective of this study is to compare spore morphology among fission yeast species.

2. The completeness of the study would be enhanced by a comparison of some of the other mutations that effect spore morphology as Reviewer 1 points out.

In response to the reviewer’s suggestion, we have added a new supplementary Table (S2 Table) showing the presence or absence of homologs of genes involved in spore membrane formation, including spo3+ and psy1+, across the fission yeast species examined in this study. We also added a corresponding description to the revised manuscript (Lines 287–288, 337).

In addition, Reviewer 1 indicated that some conclusions are overstated. Please rewrite each of these sections to accommodate these concerns.

We agree with the reviewer's comments and have revised the relevant statements throughout the manuscript to use more appropriate and cautious wording.

==============================

Journal Requirements:

We have addressed all editorial and administrative requirements requested by the journal, including funding information, data availability, code sharing, figure copyright, and reference list verification.

1. PLOS ONE formatting requirements

The manuscript and associated files have been revised to comply with PLOS ONE formatting and file-naming requirements.

2. Code sharing

We confirm that this study did not rely on any author-generated code for data analysis or generation of the reported findings. Therefore, the PLOS ONE code sharing policy is not applicable to this manuscript.

3. Funding information

We checked the grant information and corrected any inconsistencies between the Funding Information and Financial Disclosure sections.

4. Funding statement and role of funders

The Funding Statement has been revised accordingly. The funders had no role in study design, data collection and analysis, decision to publish, or preparation of the manuscript.

5. Finance disclosure

The Financial Disclosure section has been revised accordingly.

6. Data availability

We confirm that all relevant data will be made publicly available upon publication in accordance with the PLOS ONE data sharing policy.

7. “Data not shown”

We have removed all instances of the phrase “data not shown” from the manuscript. The corresponding data have been included in the Supplementary Materials, and the relevant sections of the manuscript have been revised accordingly.

8. Copyrighted figures

We have reviewed all figures and confirm that they are original works created by the authors and do not contain third-party copyrighted material.

9. Suggested references

We evaluated the suggested references and determined that no additional citations were required.

10. Reference list

We reviewed the reference list and corrected it where necessary. No retracted articles are cited in the manuscript.

Reviewers' comments:

Reviewer's Responses to Questions

Comments to the Author

Reviewer #1: This is a descriptive comparative study of the spore morphology of the four major fission yeast species. The main and interesting findings are that the morphologies are distinct, and correlate with different levels of resistance of the spores to environmental stressors. An interesting minor finding is that mating efficiency in octosporus is extremely temperature sensitive. I have no major concerns with this, and only a few minor comments.

The authors state that the protein Isp3 which coats the outside of S. pombe spores is not found in octosporus but is it found in japonicus?

We thank the reviewer for this comment. We have clarified that neither S. octosporus nor S. japonicus possesses a close homolog of Isp3 and revised the relevant text accordingly. The corresponding changes have been made in the revised manuscript (Lines 287–295).

Line 336: RNAi is known to be required for S. pombe silencing at centromeres (as written the statement implies it is not).

We agree with the reviewer. Our original statement was based on a misinterpretation of the cited reference. Therefore, we have removed the relevant text from the revised manuscript (Line 358).

In the discussion, the authors might want to speculate a bit more about the habitats of these species. pombe at least is widely dispersed in temperate as well as tropical climates. Are the others more temperate/northerly?

We thank the reviewer for this interesting suggestion. At present, there are limited data linking the distribution of fission yeast species other than S. pombe to specific climate zones. To our knowledge, previous studies have not reported whether S. japonicus or S. octosporus are preferentially associated with temperate, tropical, or northern environments. However, S. japonicus has been frequently isolated in Japan (Seike et al., 2021), suggesting that it is well established in at least some temperate regions.

In addition, Brysch-Herzberg et al., 2022 emphasized the importance of comprehensive isolation studies to identify the natural substrates from which each Schizosaccharomyces species can be most effectively recovered. Because the currently known isolation sources are geographically and environmentally biased, it remains difficult to draw firm conclusions regarding the climatic preferences of individual fission yeast species other than S. pombe. Future ecological surveys and broader sampling efforts may help clarify whether different Schizosaccharomyces species are associated with particular climate zones or habitats.

The corresponding changes have been made in the revised manuscript (Lines 370-375).

One feature not discussed much is that pombe is a 4-spored species but all the others produce 8 spores, presumably due to an extra mitosis. Could this be related to the spore morphology? Perhaps some speculation....

Whereas S. pombe typically produces four spores per ascus, S. octosporus, S. japonicus, and S. versatilis usually produce eight spores. However, asci containing only four spores are also occasionally observed in these species. Examination of these spores revealed that none exhibited the bumpy surface characteristic of S. pombe. These observations suggest that spore morphology is not related to the number of spores formed within an ascus.

The corresponding changes have been made in the revised manuscript (Lines 297–302).

Similarly, is there anything known about the rate of germination? that is, how fast after re-feeding do these spores enter vegetative growth?

The germination frequencies were similar among S. pombe (>90%), S. japonicus (88%), and S. octosporus (83%) (n > 150), indicating no substantial differences among these species. In contrast, the germination frequency of S. versatilis was approximately 20% (n > 150). However, these experiments were performed on YE medium, which may not represent the optimal growth conditions for S. versatilis. While we assessed germination frequencies, we did not measure germination kinetics. To our knowledge, no studies have systematically compared germination rates among fission yeast species. The corresponding changes have been made in the revised manuscript (Lines 303–307).

Reviewer #2: Review of PONE-26-13693, Sakaguchi et al.,

The manuscript examines the spores formed by four different, evolutionarily diverse Schizosaccharomyces species using light and electron microscopy and stress resistance. The work fills a gap in the characterization of fission yeasts, as the species examined are thought to have diverged about 200 million years ago (Mya) and the findings are quite diverse from the best studied system, S. pombe.

The manuscript is reasonably complete, but contains mistakes, presents some correlations too strongly and equivocates on more recent data shown by multiple labs. These issues are enumerated below and need to be addressed.

Major:

1) Line 67 and a new explanatory figure. The manuscript compares S. pombe, S. octosporus, S. japonicus and S. versatilis, but the evolutionary distance between these yeasts is not well presented. This reviewer suggests a figure with an evolutionary tree showing divergence of these four fission yeasts, similar to Fig. 3 in Brysch-Hershberg et al. Yeast. 2024;41:108–127. I also strongly suggest that below the yeast dendrogram and comparative dendrogram for humans and rodents such as in COMMUNICATIONS BIOLOGY | (2022) 5:986. Inclusion of an aligned panel of mammalian evolution would show that the authors are comparing yeast that diverged long before humans and rodents, and would drive home how different the yeasts are, as many labs studying mammalian systems think all yeasts are the same.

We thank the reviewer for this helpful suggestion. To clarify the evolutionary relationships among the fission yeasts examined in this study, we have added a new explanatory figure showing a phylogenetic tree of S. pombe, S. octosporus, S. japonicus, and S. versatilis, based on the recent classification and phylogenetic analysis reported by Brysch-Herzberg et al, 2024. This figure is now included as S1 Fig, and the relevant description has been added to the revised manuscript. (Line 62).

We agree that comparison with mammalian divergence would be useful for emphasizing the large evolutionary distances among fission yeasts. However, because the focus of the present study is the diversity of spore morphology within the genus Schizosaccharomyces, we have limited the new figure to the yeast species analyzed in this study. We have instead clarified in the text that these fission yeasts are evolutionarily distant species, so that readers can better appreciate that they should not be regarded as closely related variants of the same yeast model.

2) Lines 60, 117 and 271 and S. japonicus versus S. versatilis as different species. The authors equivocate as to whether the two yeasts are different species or varieties of the same species, even though they cite recent papers with whole genome sequencing and genetic data that indicates that the two yeasts are different species. Why do the authors say “suggested that…represents two different species”? Why do the authors equivocate? They should give some reason why they doubt the species reclassification.

We agree with the reviewer's comment. In accordance with recent genomic studies supporting the classification of S. japonicus and S. versatilis as distinct species, we have revised the text to avoid any ambiguity. The corresponding revisions are provided in the revised manuscript (Lines 117–118, 276-278).

3) Lines 111-182, 301-303, 353: plasma membrane structures are “highly conserved”. I take issue with the term “highly” because the figure presents plasma membranes with parallel invaginations but the structures presented look very different between species. While Saccharomyces cerevisiae has invaginations that are not parallel, the authors appear to be overstating their observation.

We agree with the reviewer's comment that the term "highly conserved" overstates our observations. To more accurately describe the results, we have revised the text to state that "the presence of parallel invaginations is conserved" among these species. The corresponding changes have been made in the revised manuscript (Lines 30, 159-160, 165, 167-168, 320-321, 378).

4) In the observation of the plasma membranes by QFDE-EM, the authors never state how many cells were observed for each species where invaginations can be judged. The authors need to present these numbers in the Results or Methods.

We agree that the number of spores examined by QFDE-EM should be stated. We have now added the number of spores analyzed for each species in the Materials and Methods section. By QFDE-EM, although the number of replicas obtained was limited because of the technical difficulty of replica preparation, we examined 9 spores of S. pombe, 9 spores of S. octosporus, 2 spores of S. japonicus, and 2 spores of S. versatilis. In addition, similar membrane invaginations were independently confirmed by transmission electron microscopy of ultrathin sections, in which 11 S. pombe, 6 S. octosporus, 11 S. japonicus, and 21 S. versatilis spores were analyzed (Fig 5). Together, these observations support the presence of membrane invaginations in all four species examined. The corresponding changes have been made in the revised manuscript (Lines 451-452, 459-463).

5) Line 205 – probably corresponds to the fibrillar structure. It is hard to know what this statement means and judge if it is true. The electron dense surface appears to be the surface of the spore, supported by the underlying electron transparent layer. The connection between the bumpy spore surface and the fibrillar adjective is not clear (I was expecting lines or cables or fibrils). The statement should either be explained or deleted.

We agree that the connection between the electron-dense outermost layer and the fibrillar surface structures was not sufficiently explained. In our previous study (Fukunishi et al., 2014), these two structures were shown to correspond to the same outermost spore wall layer. We have therefore revised the text to explicitly describe this relationship and added a reference to the corresponding QFDE-EM image (Fig 2A, lower panel). The corresponding changes have been made in the revised manuscript (Lines 207–209).

6) Lines 249-251 – The conclusion suggesting a link between stress resistance and spore wall differences is a correlation that has not been tested, and is overstated. Line 251 could state that spores are “structurally distinct and correlates with stress resistance.”

We agree with the reviewer that the previous statement was overstated. We have revised the text to clarify that the observed relationship between spore wall structure and stress resistance is a correlation. The corresponding changes have been made in the revised manuscript (Lines 254–255).

7) Lines 288-289 – The discussion of the lack of a protein layer for some spore types is based on data not shown and the absence of an electron dense layer, which is not convincing as presented. The protein gels should be presented in the Supplemental Material and the conclusion about a protein layer needs better justification. The lack of staining in the EM is not sufficient to say no protein layer exists.

We agree with the reviewer’s comment. We have now included the SDS–PAGE data in the Supplemental Material (S3 Fig). Extraction of the outermost layer of S. octosporus spores yielded two protein bands on SDS–PAGE; however, both bands were weaker and differed in molecular size from the S. pombe Isp3 protein, indicating that S. octosporus spores possess a proteinaceous surface component distinct from the Isp3 layer of S. pombe. In contrast, no distinctive protein bands were detected in S. japonicus or S. versatilis spores subjected to the same extraction procedure (S3 Fig). Based on these results, we have revised our interpretation and no longer conclude that these species completely lack an outer protein layer. Instead, we conclude that our data do not support the presence of a prominent proteinaceous outer spore wall layer comparable to the Isp3 layer of S. pombe. The corresponding revisions have been incorporated into the revised manuscript (Lines 285–295).

8) Line 326 – There are a number of genes in spore wall formation, some like spo3 and psy1 studied by the Nakamura lab, that may play a role in the different spore ultra structures.

Attachment Submitted filename: Response to Reviewers.docx

# Peer review: Decision Letter 1

**DOI:** 10.1371/journal.pone.0355697.r003

**Authors:** Arthur Lustig

13 Jul 2026

Dear Dr. Nakamura,

Thank you for submitting your manuscript to PLOS ONE. After careful consideration, we feel that it has merit but does not fully meet PLOS ONE’s publication criteria as it currently stands. Therefore, we invite you to submit a revised version of the manuscript that addresses the points raised during the review process.

plosone@plos.org. When you're ready to submit your revision, log on to https://www.editorialmanager.com/pone/ and select the 'Submissions Needing Revision' folder to locate your manuscript file.

- A letter that responds to each point raised by the academic editor and reviewer(s). You should upload this letter as a separate file labeled 'Response to Reviewers'.
- A marked-up copy of your manuscript that highlights changes made to the original version. You should upload this as a separate file labeled 'Revised Manuscript with Track Changes'.
- An unmarked version of your revised paper without tracked changes. You should upload this as a separate file labeled 'Manuscript'.

If you would like to make changes to your financial disclosure, please include your updated statement in your cover letter. Guidelines for resubmitting your figure files are available below the reviewer comments at the end of this letter.

If applicable, we recommend that you deposit your laboratory protocols in protocols.io to enhance the reproducibility of your results. Protocols.io assigns your protocol its own identifier (DOI) so that it can be cited independently in the future. For instructions see: https://journals.plos.org/plosone/s/submission-guidelines#loc-laboratory-protocols. Additionally, PLOS ONE offers an option for publishing peer-reviewed Lab Protocol articles, which describe protocols hosted on protocols.io. Read more information on sharing protocols at https://plos.org/protocols?utm_medium=editorial-email&utm_source=authorletters&utm_campaign=protocols.

As the corresponding author, your ORCID iD is verified in the submission system and will appear in the published article. PLOS supports the use of ORCID, and we encourage all coauthors to register for an ORCID iD and use it as well. Please encourage your coauthors to verify their ORCID iD within the submission system before final acceptance, as unverified ORCID iDs will not appear in the published article. *Only*  the individual author can complete the verification step; PLOS staff *cannot*  verify ORCID iDs on behalf of authors.

We look forward to receiving your revised manuscript.

Kind regards,

Arthur J. Lustig, PhD

Academic Editor

PLOS One

Journal Requirements:

1. If the reviewer comments include a recommendation to cite specific previously published works, please review and evaluate these publications to determine whether they are relevant and should be cited. There is no requirement to cite these works unless the editor has indicated otherwise.

2. Please review your reference list to ensure that it is complete and correct. If you have cited papers that have been retracted, please include the rationale for doing so in the manuscript text, or remove these references and replace them with relevant current references. Any changes to the reference list should be mentioned in the rebuttal letter that accompanies your revised manuscript. If you need to cite a retracted article, indicate the article’s retracted status in the References list and also include a citation and full reference for the retraction notice.

[Note: HTML markup is below. Please do not edit.]

Reviewers' comments:

Reviewer's Responses to Questions

**Comments to the Author**

Reviewer #2: (No Response)

**********

2. Is the manuscript technically sound, and do the data support the conclusions?

Reviewer #2: Yes

**********

3. Has the statistical analysis been performed appropriately and rigorously?

Reviewer #2: N/A

**********

4. Have the authors made all data underlying the findings in their manuscript fully available?

The PLOS Data policy

Reviewer #2: Yes

**********

5. Is the manuscript presented in an intelligible fashion and written in standard English?

Reviewer #2: Yes

**********

Reviewer #2: Please see the attached PDF for my review and the modified Fig. S1 that shows my earlier suggestion for comparing Schizosaccharomyces and mammalian evolution.

**********

what does this mean?). If published, this will include your full peer review and any attached files.

If you choose “no”, your identity will remain anonymous but your review may still be made public.

**Do you want your identity to be public for this peer review?**  For information about this choice, including consent withdrawal, please see our Privacy Policy

Reviewer #2: No

**********

[NOTE: If reviewer comments were submitted as an attachment file, they will be attached to this email and accessible via the submission site. Please log into your account, locate the manuscript record, and check for the action link "View Attachments". If this link does not appear, there are no attachment files.]

To ensure your figures meet our technical requirements, please review our figure guidelines: https://journals.plos.org/plosone/s/figures

You may also use PLOS’s free figure tool, NAAS, to help you prepare publication quality figures: https://journals.plos.org/plosone/s/figures#loc-tools-for-figure-preparation.

NAAS will assess whether your figures meet our technical requirements by comparing each figure against our figure specifications.

Attachment Submitted filename: Review_of_PONE-D-26-13693R1.pdf

# Author comment: Author response to Decision Letter 2

**DOI:** 10.1371/journal.pone.0355697.r004

21 Jul 2026

We thank Reviewer 2 for the thoughtful and constructive comments.

1. Comparison with mammalian evolution

Reviewer’s comment:

I think the comparison to mammalian evolution is an important point to be included, and the figure does not have to be a complete dendrogram with humans and fission yeasts. It is important because, especially with the rise conservative governments and the attitudes towards basic research compared to applied biomedical research, the profound nature of the Schizosaccharomyces species divergence will be overlooked if it is not driven home. I have multiple, personal examples of anthropocentric researchers claiming that a protein is conserved across mammals is important when that conservation is insignificant. This issue will persist and grow long after the senior author of this manuscript, this reviewer and the editor have been forced out of science. This figure has the opportunity to make a slide that can be used by many fission yeast researchers in the future to show how useful these comparisons are for protein and cellular evolution. I will add that my colleagues in the budding yeast community had to make similar arguments when discussing the evolutionary divergence of Saccharomyces species decades ago. The divergence of Schizosaccharomyces species is even more ancient, so the evolutionary comparisons are more meaningful.

I am providing a modified version of Figure S1 in this pdf to explain what I mean, including references not intended for a final figure. Briefly, small labels show when humans diverged from other mammals at the bottom of the main fission yeast dendrogram, so one can use this as a guide for the evolutionary time scale. (I will leave it to future users to add little silhouettes or illustrations for these evolutionary landmarks.) The bottom of the figure includes a time scale I made to place the tick marks and the references use to give the divergence times from present day (humans-cows 85 Mya, humans-mice 92 Mya, humans-kangaroos(marsupials) 148 Mya, humans-platypus(monotremes) 166 Mya, first mammalian fossil 225 Mya, first reptile 318 Mya) so the authors can judge the figure before making their own version.

A remaining open question is whether early pre-humans made pombe beer or used the other

Response:

We greatly appreciate the reviewer’s detailed explanation and the modified version of Figure S1 provided as a helpful example. Following the reviewer’s suggestion, we prepared a revised phylogenetic tree using the proposed tree and cited references as guides. We also revised the legend of Figure S1 accordingly. In addition, the three papers cited in the reviewer’s annotated figure have now been added to the References.

2. The completeness of the study would be enhanced by a comparison of some of the other mutations that effect spore morphology as Reviewer 1 points out.

Reviewer’s response:

The authors’ response satisfies my concern.

Response:

We thank the reviewer for confirming that our previous response satisfactorily addressed this concern.

Issues and typographical errors:

1. Lines 238-239 vs. Fig. S2A: “that of S. octosporus increased with decreasing temperature, reaching ~70% at both 20°C and 15°C. S. versatilis exhibited the highest mating rate (~70%) at 30°C, with a gradual decline at lower temperatures” I think that S. octosporus (So) and S. versatilis (Sv) are switched in this sentence, or the Fig2A bar graph is mislabeled. In my S2_fig.tif file downloaded with the revised manuscript, the frequency of So sporulation is lowest at 15 C and highest at 30C. The Sv sporulation frequency is highest at 15 C while 25 and 20 C are similar. The rest of the paragraph should be checked for accuracy.

Response:

We carefully re-examined both the text and Figure S2A and confirmed that the description in the text was correct, whereas Figure S2A contained an error. Specifically, the temperature labels corresponding to the graph colors were incorrect. We have now corrected these labels.

In responding to the reviewer’s previous comment, we mistakenly understood that the comment referred to the graph in Figure 6 and therefore did not revise Figure S2A at that time. We apologize for this oversight.

2. Lines 306-307: “these fission yeast spores did not fluoresce under ultraviolet light, a

characteristic feature of the dityrosine layer (S4 Fig).

Response:

We checked the original manuscript and confirmed that the citation in the text itself was correct. However, Figures S3 and S4 had inadvertently been submitted in the reverse order. We have now corrected the order of these figures.

3. Line 340: “included within the limited set of 100–400 non-conserved genes in fission

yeasts”

The authors should provide a reference or references for where these numbers come from. I am guessing that these are summaries from the genome sequences of the different species compared with S. pombe but it would be helpful to others to give the basis for this statement.

Response:

We agree that the basis for this statement should be clearly documented. We have therefore added the relevant references to support the estimate of 100–400 non-conserved genes. We also added appropriate references to the preceding sentence for further clarification. (Lines 330, 340).

4. Lines 602-604 and Fig. S5 legend: “The mean gray values of the regions indicated by

arrows”

It took me some time to interpret this figure. I believe that the “gray area” in the pipet tip is

spores that adhere to the tip, showing the hydrophobic interaction.

Please add a sentence to the legend for Fig. S5A explaining that the gray area are spores

that remain adhered to the pipet tip. The Methods section does not describe how this test was done. A brief explanation could be in either the Methods or the Fig. S5 legend.

Response:

As suggested by the reviewer, we expanded the legend of Figure S5A to include a brief description of the experimental procedure. We also clarified that the gray regions indicated in the pipette tip correspond to spores that remained attached to the inner surface of the tip.

Attachment Submitted filename: Response_to_Reviewers_auresp_2.docx

# Editor report: Decision Letter 2

**DOI:** 10.1371/journal.pone.0355697.r005

**Authors:** Arthur Lustig

26 Jul 2026

Diversity of Spore Morphology among Fission Yeasts

PONE-D-26-13693R2

Dear Dr. Nakamura,

We’re pleased to inform you that your manuscript has been judged scientifically suitable for publication and will be formally accepted for publication once it meets all outstanding technical requirements.

Within one week, you’ll receive an e-mail detailing the required amendments. When these have been addressed, you’ll receive a formal acceptance letter and your manuscript will be scheduled for publication.

An invoice will be generated when your article is formally accepted. Please note, if your institution has a publishing partnership with PLOS and your article meets the relevant criteria, all or part of your publication costs will be covered. Please make sure your user information is up-to-date by logging into Editorial Manager at Editorial Manager® and clicking the ‘Update My Information' link at the top of the page. For questions related to billing, please contact billing support.

If your institution or institutions have a press office, please notify them about your upcoming paper to help maximize its impact. If they’ll be preparing press materials, please inform our press team as soon as possible -- no later than 48 hours after receiving the formal acceptance. Your manuscript will remain under strict press embargo until 2 pm Eastern Time on the date of publication. For more information, please contact onepress@plos.org.

Kind regards,

Arthur J. Lustig, PhD

Academic Editor

PLOS One

Additional Editor Comments (optional):

Reviewers' comments:

# Editor report: Acceptance letter

**DOI:** 10.1371/journal.pone.0355697.r006

**Authors:** Arthur Lustig

PONE-D-26-13693R2

PLOS One

Dear Dr. Nakamura,

I'm pleased to inform you that your manuscript has been deemed suitable for publication in PLOS One. Congratulations! Your manuscript is now being handed over to our production team.

At this stage, our production department will prepare your paper for publication. This includes ensuring the following:

* All references, tables, and figures are properly cited

* All relevant supporting information is included in the manuscript submission,

* There are no issues that prevent the paper from being properly typeset

You will receive further instructions from the production team, including instructions on how to review your proof when it is ready. Please keep in mind that we are working through a large volume of accepted articles, so please give us a few days to review your paper and let you know the next and final steps.

Lastly, if your institution or institutions have a press office, please let them know about your upcoming paper now to help maximize its impact. If they'll be preparing press materials, please inform our press team within the next 48 hours. Your manuscript will remain under strict press embargo until 2 pm Eastern Time on the date of publication. For more information, please contact onepress@plos.org.

You will receive an invoice from PLOS for your publication fee after your manuscript has reached the completed accept phase. If you receive an email requesting payment before acceptance or for any other service, this may be a phishing scheme. Learn how to identify phishing emails and protect your accounts at https://explore.plos.org/phishing.

If we can help with anything else, please email us at customercare@plos.org.

Thank you for submitting your work to PLOS ONE and supporting open access.

Kind regards,

PLOS ONE Editorial Office Staff

on behalf of

Dr. Arthur J. Lustig

Academic Editor

PLOS One
